# Reconstructing reactivity in dynamic host–guest systems at atomistic resolution: amide hydrolysis under confinement in the cavity of a coordination cage†

**DOI:** 10.1039/d2sc02000a

**Published:** 2022-08-29

**Authors:** Massimo Delle Piane, Luca Pesce, Matteo Cioni, Giovanni M. Pavan

**Affiliations:** Department of Applied Science and Technology, Politecnico di Torino Corso Duca degli Abruzzi 24 10129 Torino Italy giovanni.pavan@polito.it; Department of Innovative Technologies, University of Applied Sciences and Arts of Southern Switzerland, Polo Universitario Lugano Campus Est, Via la Santa 1 6962 Lugano-Viganello Switzerland

## Abstract

Spatial confinement is widely employed by nature to attain unique efficiency in controlling chemical reactions. Notable examples are enzymes, which selectively bind reactants and exquisitely regulate their conversion into products. In an attempt to mimic natural catalytic systems, supramolecular metal–organic cages capable of encapsulating guests in their cavity and of controlling/accelerating chemical reactions under confinement are attracting increasing interest. However, the complex nature of these systems, where reactants/products continuously exchange in-and-out of the host, makes it often difficult to elucidate the factors controlling the reactivity in dynamic regimes. As a case study, here we focus on a coordination cage that can encapsulate amide guests and enhance their hydrolysis by favoring their mechanical twisting towards reactive molecular configurations under confinement. We designed an advanced multiscale simulation approach that allows us to reconstruct the reactivity in such host–guest systems in dynamic regimes. In this way, we can characterize amide encapsulation/expulsion in/out of the cage cavity (thermodynamics and kinetics), coupling such host–guest dynamic equilibrium with characteristic hydrolysis reaction constants. All computed kinetic/thermodynamic data are then combined, obtaining a statistical estimation of reaction acceleration in the host–guest system that is found in optimal agreement with the available experimental trends. This shows how, to understand the key factors controlling accelerations/variations in the reaction under confinement, it is necessary to take into account all dynamic processes that occur as intimately entangled in such host–guest systems. This also provides us with a flexible computational framework, useful to build structure–dynamics–property relationships for a variety of reactive host–guest systems.

## Abstract 2

Encapsulation of guests in metal–organic cages allows control over chemical reactivity. Focusing on the hydrolysis of amides, here we show an effective molecular simulation approach to reconstruct reactivity in host–guest systems in dynamic regimes.

## Graphical abstract

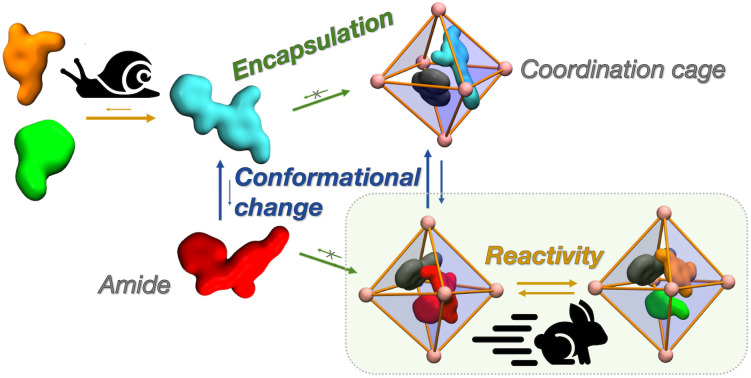

## 1 Introduction

In billions of years of evolution, nature has evolved systems (or materials) to control chemical reactivity with unique specificity, efficiency and fidelity.^1,2^ Enzymes, capable of catalyzing reactions in their substrate-binding cavities, are a notable example.^3,4^ In these systems, the reactants are dynamically encapsulated in the enzyme binding site, where the reaction occurs, and the products are then expelled leaving the reaction site free for hosting another reaction.^5^ Such machinery is controlled by a delicate modulation of host–guest interactions, which controls key factors, such as, *e.g.*, the residence time of the reactants/products (guest) in the reaction site of the enzymes (host), conformational changes in the guests favoring the reaction, *etc.*, controlling *de facto*, the reactivity in the system.^6–8^

In an attempt to mimic natural catalytic systems, chemists have designed synthetic cavities capable of encapsulating reactants with high selectivity.^9–11^ In particular, supramolecular coordination cages have been designed, which can host catalytic reactions in their internal cavities.^10,12–15^ Reactivity, regio-selectivity and enantio-selectivity can be manipulated in such systems, *e.g.* by engineering the structural and electronic properties of the cage frameworks or by changing the guest structure.^9,16^ Noteworthily, by mimicking what happens in some proteolytic enzymes,^17^ in a recent study it has been shown that the encapsulation of amide guests in a coordination cage may result in a considerable acceleration of amide hydrolysis. In particular, the molecular crowding in the cavity of the cage was found to favor the mechanical twisting of the amides towards reactive (*cis* isomers) configurations.^10^ The rational design of similar synthetic molecular systems requires a detailed comprehension of (i) the molecular and chemical–physical factors that control their reactivity in space and time, and (ii) how to master them in order to produce new classes of catalytic systems. However, in such supramolecular host–guest systems, the reactivity is coupled with a dynamic equilibrium where the guests exchange in/out of the cage hosts, which makes them difficult to rationalize.^16,18,19^

In general, the reactivity in such systems is controlled by several factors, such as guest binding/unbinding, the solubility of the guests in the outer solution, the characteristic timescale for the reactions, possible entrapment in metastable states, and molecular concentrations in the system. In the presence of coordination-cages, a major role in reactivity is played by non-covalent cavity-guest interactions.^10,16,20^

Despite notable efforts,^9,15,20–22^ reaching a detailed understanding in terms of the molecular factors that control the reactivity in systems in which the molecular species are in continuous exchange is typically difficult at the experimental level. Computer simulations are extremely useful to this end. Quantum mechanical (QM) approaches^23^ or semi-empirical approaches such as density functional tight binding (DF-TB) methods ^24^ have been employed to study chemical reactivity and reactive pathways with notable precision. *Ab initio* molecular dynamics (MD)^25–27^ and metadynamics^28–30^ simulations have been also widely adopted to study chemical reactions of reactants in reactive configurations, in cases where the reactions require crossing free-energy barriers. However, the study of such systems on timescales that also allows accounting for their supramolecular host–guest dynamics (*i.e.*, the dynamic exchange of guests in-and-out of the host) is not trivial. For this reason, a comprehensive description of such complex dynamic systems has been difficult to attain until now. Recent computational approaches based on enhanced sampling methods, such as, *e.g.*, metadynamics (MetaD), hold great potential in this sense.^16,31–33^ Such recently employed approaches, *e.g.*, to study the isomerization of photochromic switches (azobenzenes) encapsulated in coordination cages, allowed the demonstration of the tight interplay between isomerization dynamics, molecular crowding, and host–guest exchange dynamics.^16^ This approach is versatile and holds high potential for studying chemical reactions in host–guest systems in dynamic regimes in general.

**Fig. 1:**
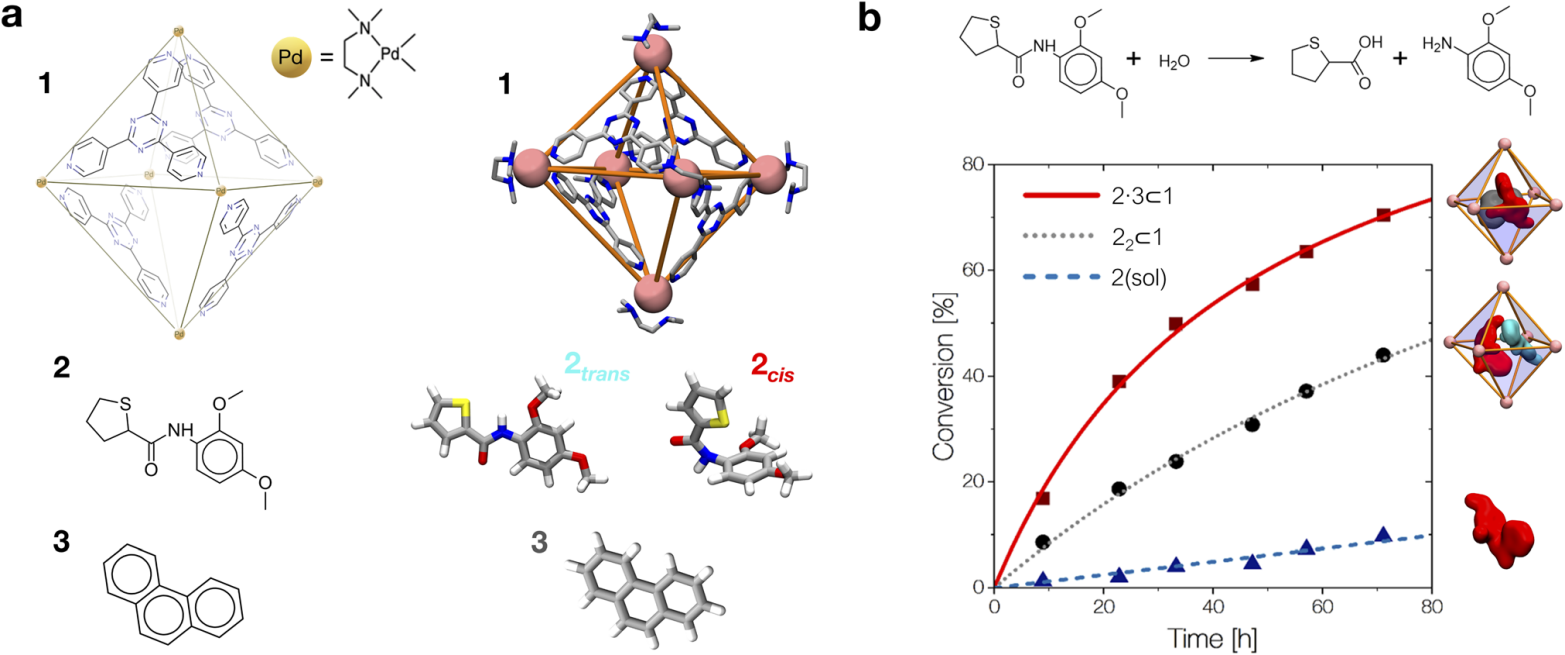
The host–guest systems studied in this work. (a, top) The octahedral coordination cage 1 used as a reference in this work is composed of four panel ligands (2,4,6-tris(4-pyridyl)-1,3,5-triazine) and six metal corners (*cis*-endcapped Pd(ii) complexes): chemical structure on the left and AA model on the right. (a, bottom) Chemical structure of amide guest 2 and of pirine co-guest 3. AA models are reported on the right of each guest structure (*cis* and *trans* isomers are shown for 2). (b, top) The hydrolysis reaction of the amide bond of 2. (b, bottom) Experimentally observed percentage of hydrolyzed 2 over time. Conversion data are reported for the hydrolysis of 2 free in solution (in blue), in the case when two 2 guests are co-encapsulated in cage 1 (black), and for 2 co-encapsulated with co-guest 3 in cage 1 (in red: ∼14× hydrolysis acceleration compared to the blue curve).^10^

As a case study, here we focus on the neat host–guest system recently reported by the group of Fujita,^10^ where the encapsulation of amide guests within a coordination cage was found to considerably enhance the amide hydrolysis. This is an interesting test case not only because one amide functional group (the peptide bond) is the structural foundation of proteins, but also because the hydrolysis of amides is a classic example of a spontaneous reaction hindered by very high kinetic barriers, which can be lowered by physical means.^34^ X-ray crystallography and NMR measurements were used in this system to characterize the encapsulation of a number of electron-rich diaryl amides into different octahedral coordination cages, differing in their metal corners and having electron-deficient walls. In particular, here we focus on the host–guest system shown in Fig. 1, for which an acceleration in the amide hydrolysis of ∼14× (compared to the same amide free in solution) has been experimentally reported when amide 2 is co-encapsulated with co-guest cage 3 inside cage 1 (Fig. 1b: red *vs.* blue curves). This has been imputed to the fact that reactive conformations of the amide guest (*cis*-twisted) are stabilized within the cage host.^10^ Such evidence is found to be consistent with single-crystal X-ray diffraction analyses, showing that the amide can twist when encapsulated within the cage cavity.^10^

To obtain a submolecular-resolution insight into the behavior of such a system in dynamic regimes, here we devised a comprehensive multiscale computational strategy. This includes the following key steps: (i) all-atom (AA) MD simulations to characterize the (structural and dynamical) features of the host–guest system under equilibrium conditions; (ii) AA metadynamics (MetaD) simulations to investigate which guest conformers are more/less favored in the cage cavity or in solution; (iii) *ab initio* MetaD simulations to estimate the relative reactivity of the various conformations that the amide guest can assume (*i.e.*, which amide conformations are more reactive). Altogether, (ii) + (iii) provide statistical information on the relative probability of stabilizing reactive guest conformers in the cage cavity. Then, in phase (iv), we conduct AA MetaD to study the host–guest equilibrium and to reconstruct the probability of effectively having the guest encapsulated in the cage cavity, the guest encapsulation residence time, *etc.* Altogether, (ii) + (iii) + (iv) provide information on the probability of effectively having reactive amide conformers encapsulated in the cage cavity. Finally, in phase (v), all data are then combined in order to obtain a probabilistic estimation of reaction acceleration (a) in the host–guest systems, which essentially compares the probability of having reactive amide conformers within the cage *vs.* in solution. We obtain results that are found in excellent agreement with the available experimental trends, as well as a flexible computational framework, which can be used, in principle, to study a variety of dynamically reactive host–guest systems and to draw structure–dynamics–reactivity relationships useful for rational design.

## 2 Results and discussion

### 2.1 Atomistic modeling of the host–guest system

As a representative example of a supramolecular host, here we focus on the coordination cage 1 reported in Fig. 1a recently employed by Takezawa *et al.* to host the hydrolysis of encapsulated amide guests.^10^ This is an octahedral coordination cage composed of four self-assembled electron-deficient panel ligands (2,4,6-tris(4-pyridyl)-1,3,5-triazine) and six metal-based corners (*cis*-endcapped Pd(ii) complexes).^35^ As a first step, starting from the X-ray crystal structure reported in the literature,^10^ we built an AA model for cage 1 which was then preliminarily minimized and equilibrated in explicit water solvent and under standard (room) conditions of temperature and pressure *via* a classical MD simulation. In particular, all the AA models used herein have been parametrized based on the general AMBER force field (GAFF)^36^ and all simulations have been run with the GROMACS-2020.2 software^37^ patched with Plumed-2.7 ^38^ (details are available in the Computational methods section in the ESI†). We analyzed the equilibrium MD trajectories to study the degree of flexibility of the cage under realistic conditions. Analysis of the evolution along with the MD of, *e.g.*, the internal volume of the cage cavity and root mean square deviation (RMSD) of the atomic positions and of two variables (d1 and d2) estimating respectively the height and equatorial width of the octahedral cage revealed that the cage structure is rather rigid under experimentally relevant conditions (see Fig. S2 in the ESI†). This is due to the tetrahedral *T*_d_ symmetry of this cage. Different from other examples of flexible cage hosts,^16^ cage 1 thus shows a large open volume (∼1.2 nm^3^), with a rather persistent hydrophobic cavity that allows encapsulating one or multiple guest molecules in its interior (the electron-acceptor π planes of the triazine-based ligands of 1 are particularly apt at interacting with electron rich guests).^10,35^ As the main guest, here we focus on guest 2, *N*-(2,4-dimethoxyphenyl)thiophene-2-carboxamide, an electron-rich diaryl amide (Fig. 1a) that was experimentally shown to produce considerable reaction acceleration following encapsulation in cage 1 (Fig. 1b).^10^ The central bond in amide guest 2 can undergo hydrolysis according to the reaction schematized in Fig. 1b (top). Guest 2 is mildly apolar (log *P* = 2.77).^39^ Experimentally obtained crystal structures show that cage 1 can encapsulate at the same time up to two 2 molecules in its internal cavity.^10^ An AA model was developed for guest 2, paying particular attention to the accuracy in the force field parametrization of the central amide bond dihedral, which defines the *trans* and *cis* conformers of the amide, how much one is energetically favored with respect to the other, and the related transition barrier. To achieve this, we optimized the original amide *ω* dihedral force field parameters of the GAFF, in order to obtain a *trans*-to-*cis* isomerization free energy profile consistent with the experimental data available for *N*-metyl-acetamide (NMA, see Fig. S1 and extended details in the Methods section in the ESI†).^40^

We built inclusion complexes where guest 2 is encapsulated within cage 1 in different stoichiometries: *i.e.*, 2 ⊂ 1 and 2_2_ ⊂ 1, where respectively one or two 2 amide guests are encapsulated inside cage 1. In particular, the AA model for the 2_2_ ⊂ 1 complex was built starting from the available experimental crystal structure for this complex,^10^ while that for the 2 ⊂ 1 complex was obtained by the deletion of one of the 2 guests.

**Fig. 2:**
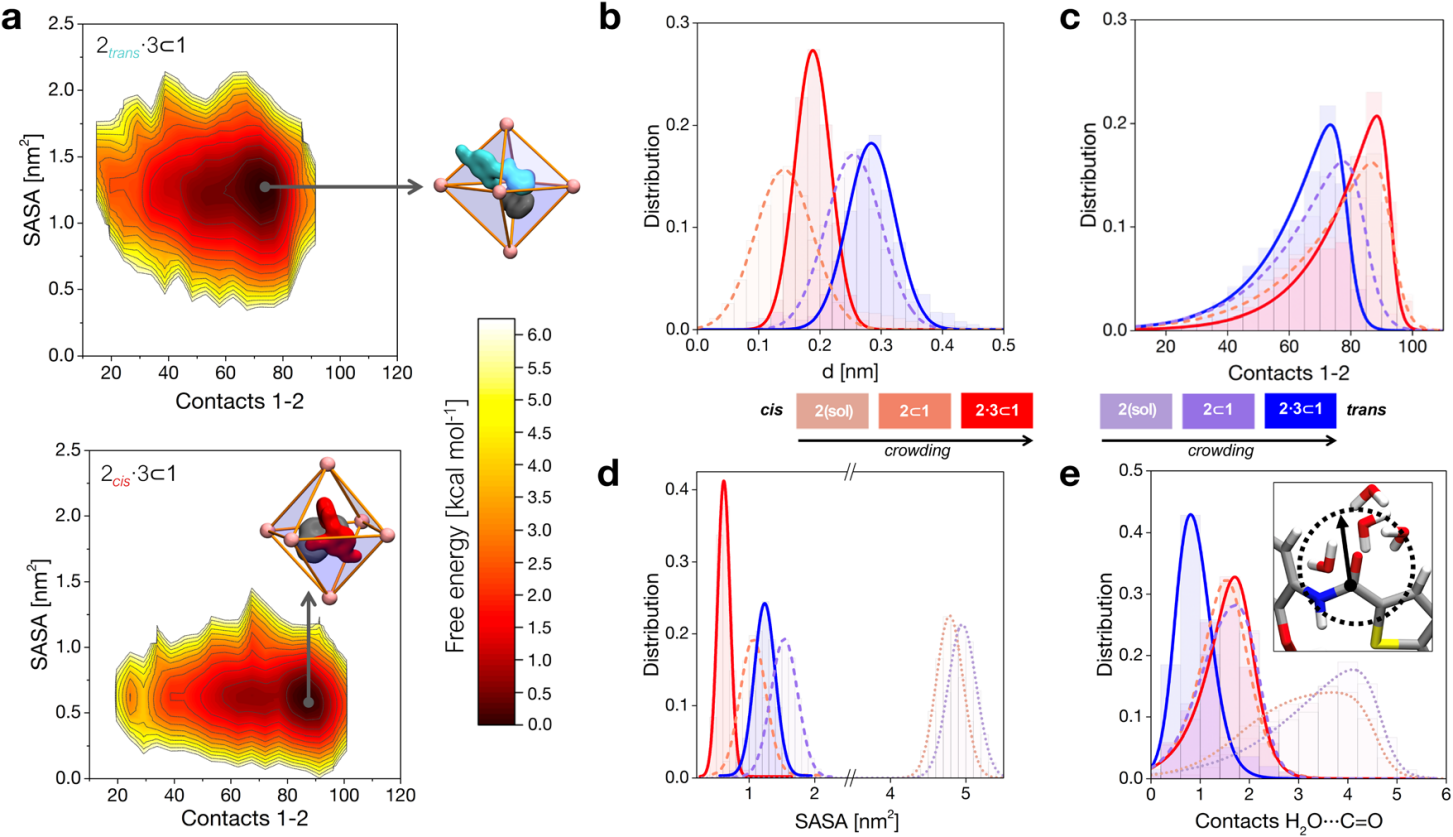
Insights into the host–guest complexes at atomistic resolution. (a) Free-energy surfaces (FESs) computed from the equilibrium MD trajectories, showing the most favorable complex configurations as a function of the contacts between 1 and 2 (*x* axis) and of the solvent accessible surface srea (SASA) of 2 within the cage (*y* axis). FESs are reported respectively for 2_*trans*_ (top) and 2_*cis*_ (bottom) co-encapsulated with co-guest 3 in the cage. For each case, a representative scheme is shown of the encapsulated structures. (b) Histogram calculated from the equilibrium MD trajectories of the distance between the geometric center of cage 1 and the center of 2. (c) Histogram of the contacts between 1 and 2. (d) Histogram of the SASA of 2 under different complexation conditions (dotted distributions at large SASA values for the free guests in solution are reported for comparison). (d) Number of contacts between the oxygen atom in the amide of 2 and the water molecules in system. A color scale is used in panels (b–e) to show increasing crowding conditions for 2 within the cage.

Aromatic amides, like 2, exist mainly in a *trans*-planar conformation in solution. However, experimental evidence demonstrated that 2 may adopt a *cis*-twisted conformation within the *T*_d_ symmetric cavity (forming a pseudo-*S*_4_ symmetric dimer) in cage 1.^10^ In particular, X-ray and NMR measurements showed signals corresponding to a *cis* : *trans* 1 : 1 2 dimer in the 2_2_ ⊂ 1 complex, and to a *cis*-twisted conformation in the 2·3 ⊂ 1 complex. For the purpose of our investigation, here we decided to model all possible combinations of conformers, mainly aiming to explore the presence of any correlation between confinement, crowding and the rotation of the amide bond. We also parametrized an AA model for co-guest 3 (Fig. 1a). Starting also in this case from a corresponding experimentally available crystal structure,^10^ we use this to build an additional AA model for the 2·3 ⊂ 1 complex, a ternary inclusion complex where one 2 guest and one 3 co-guest are simultaneously encapsulated in cage 1. Particularly interesting for this study is that the 2·3 ⊂ 1 system was observed experimentally to produce the largest hydrolysis acceleration among all explored cases (Fig. 1b: ∼14× acceleration compared to that of the free amide).^10^ We then ran classical MD simulations to equilibrate all the considered complexes in explicit water and under standard (room) conditions of temperature and pressure for at least 1 μs for each system, which was found to guarantee sufficient sampling for all variables reported in Fig. 2, see S3 and the ESI† for details). From the equilibrium MD trajectories, we then extracted multiple data indicative of the encapsulation (Fig. 2).

When in the cage, 2 tends to stay shifted from the geometrical center of the cavity to maximize interactions with the walls, with the 2_*trans*_ conformer showing in general a larger shift compared to 2_*cis*_ (Fig. 2b). This is imputable to the thiophene ring, which tends to partially stick out of the cavity preferring interaction with the solvent (scheme of Fig. 2a, top: in cyan). Co-encapsulation with 3 induces a larger decentralization of the amide guests. Noteworthily, the augmented growth in such a case is observed to cause a significant reduction in terms of 2_*cis*_ mobility in the cage cavity, as evidenced by the narrower distribution in Fig. 2b (solid red curve). This behavior correlates with an augmented number of contacts between 2 and cage 1 (Fig. 2c). In general, the 2_*cis*_ guest shows an increased number of contacts than the 2_*trans*_ one (augmented guest-cage interaction), while this trend is even increased when co-guest 3 is also present within 1 (higher crowding). We will discuss in the next section how this affects which conformer (2_*trans*_*vs.*2_*cis*_) is more favored within the cage in different complexes.

The solvent accessible surface area (SASA) of the guests shows that 2 is less exposed to the solvent when this is encapsulated within 1, and even less when this is co-encapsulated together with 3 (Fig. 2d). In general, we observe that the 2_*cis*_ conformer has a smaller SASA, and is more compact than 2_*trans*_ in all cases. These SASA and contact data provide information on different packings of the 2_*cis*_ and 2_*trans*_ conformers within the cage under increasing crowding conditions. These are consistent with the free-energy surfaces (FESs) reconstructed from the histograms extracted from the MD shown in Fig. 2a, where it is evident how 2_*cis*_ allows for a tighter packing within the cage (bottom). At the same time, the 2_*cis*_ conformer is found to be less mobile within 1, as it is demonstrated by a narrower FES dark minimum (minimum energy configuration) compared to that of the 2_*trans*_ conformer (top).

As a first and generally rate-limiting step, the amide hydrolysis reaction requires the nucleophilic attack of water (or of OH^−^, if the reaction occurs as catalyzed under basic conditions) on the carbonyl carbon.^34,41^ While the cavity of cage 1 is markedly apolar and the encapsulation of the guests in the cavity is essentially driven by hydrophobic effects, some accessibility by the solvent is therefore still required for the reaction to take place. We calculated the number of contacts between the solvent molecules and the carbonyl group of the 2 guest in various cases. In general, we can observe that the amide of 2_*trans*_ is slightly more accessible to the solvent compared to that of 2_*cis*_ when the guests are free in solution (dotted distributions), and a similar depletion is observed upon mono-guest confinement (dashed). However, the situation is surprisingly switched in the tightly packed 2·3 ⊂ 1 systems (Fig. 2e). Under highly confined conditions, the amide of 2_*cis*_ is found to be more exposed to the solvent within cage 1 compared to that of 2_*trans*_ (red *vs.* blue solid curves). This suggests that, while on the one hand the *cis* conformer of 2 is more tightly packed within the cage, the equilibrium configuration of 2_*cis*_ in the 2·3 ⊂ 1 complex may allow, at the same time, for an increased propensity to react.

### 2.2 Relationship between amide conformations and reactivity

We explore at a deeper level the difference in terms of reactivity between the *trans vs. cis* conformers of 2. We do this by decomposing the study into two phases. First, by means of AA MetaD simulations, we compute the free energy landscape for all conformers accessible by the guests in *vs.* out of the cage, obtaining information on the probability of effectively visiting and observing reactive guest conformers in the cage cavity *vs.* in solution. Then, by means of *ab initio* MetaD simulations we estimate the relative hydrolysis reactivity of the different accessible conformations the guest can assume (*i.e.*, characterized by different *ω* values) in or out of the cage cavity.

Under realistic conditions, amide bonds are usually found in *trans* configurations with a torsion angle *ω* close to π, with a sparse population in *cis* conformation (*ω* ∼ 0). The degree of steric conflict of the two residues flanking the amide bond is typically larger in *cis* amides, resulting, for example, in only ∼5–6% occurrence of *cis* peptide bonds in protein structures.^42,43^ To estimate the relative probabilities for finding different conformers under different conditions, we used WT-MetaD simulations, providing free energy profiles of the *ω* isomerization.

**Fig. 3:**
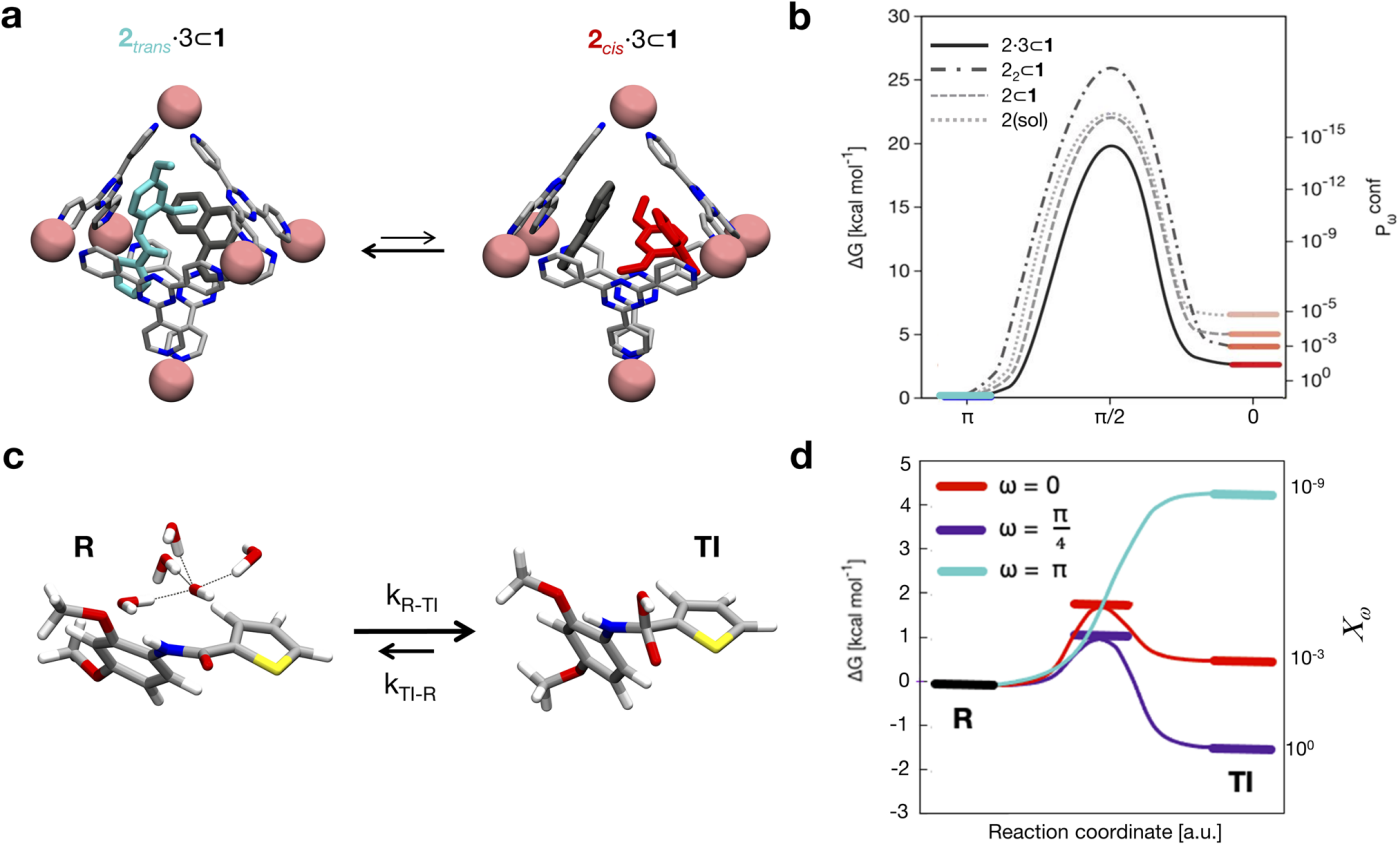
Conformers and reactivity of 2. (a) Isomerization of 2 in the cage, co-encapsulated with 3 (left: *trans*-2, right: *cis*-2). (b) Free energy profiles (shown as smoothed fits between the computed critical points) for the isomerization of 2 (i) when it is free in solution (dotted curve, *cis* in pink), (ii) when 2 is encapsulated in 1 (dashed curve, *cis* in dark pink), (iii) when it is co-encapsulated with another (*trans*) 2 guest in cage 1 (dot-dashed curve, *cis* in light red), and (iv) when 2 is encapsulated in 1 together with the co-guest 3 (solid curve, *cis* state in light red). The data show that increasing crowding stabilizes more and more of the reactive 2 conformations (*e.g.*, *cis*) in the cage cavity. Right secondary *y* axis: relative probabilities (*P*^conf^_*ω*_) of the different conformations (*ω*) of 2 in the various host–guest systems calculated based on the Δ*G* values extracted from WT-MetaD simulations. (c) Reaction scheme modelling the first step of the hydrolysis, where the oxydrile anion (OH^−^) approaches the amide group (state R) attacking the carbonyl group (formation of the transition intermediate TI). (d) Schemes of free energy profiles (shown as smoothed fits between the computed critical points) for oxydrile attack along the reaction coordinate as a function of the *ω* dihedral of 2 different values. The cyan profile refers to 2 in *trans* conformation, the red one refers to the reaction when 2 is *cis*, while the violet profile refers to the free energy profile of a *cis*-distorted configuration of 2 with *ω* = π/4. A relative reactivity score for each amide conformer (*χ*_*ω*_), normalized based on the maximum measured value (*i.e.*, that for *ω* = π/4, set to 1), is associated with the simulated conformers of 2 (right secondary *y* axis).

The *trans*-to-*cis* isomerization of *ω* angles consists of a local conformation change that is often compensated by local variations of the backbone angles *ϕ* and *ψ* of the residues flanking the amide.^42^ In order to assess how the free energy profile for the isomerization of *ω* is affected by the torsion of *ψ* and *ϕ*, we selected these 3 dihedral angles as our CVs and ran WT-MetaD simulations activating/biasing the *trans*-to-*cis* transition of 2 (*i.e.*, the torsion around the amide bond) under different conditions (Fig. 3a and b). Preliminary WT-MetaD simulations showed that the free energy profile of the *ω* isomerization is not particularly influenced by the *ϕ* and *ψ* torsions. Well-converged WT-MetaD runs allowed us to reconstruct the differences in free energies between the conformers (Δ*G*) and estimate the free energy barriers (to this end we used infrequent WT-MetaD simulations, as recrossing WT-MetaD simulations may underestimate the barrier heights – see extended Methods for details) (Table 1).

**Table 1:** Thermodynamic and kinetic data for 2 isomerization in all simulated complexes. Free energy differences (Δ*G*_*trans*→*cis*_) related to the *trans*-to-*cis* isomerization, equilibrium constants for the conformational change (*K*_conf_), the height of the free-energy barriers 
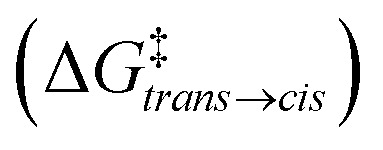
 from the *trans* state, and characteristic timescales (*t*_*trans*→*cis*_ and *t*_*cis*→*trans*_) are reported

Process	Δ*G*_*trans*→*cis*_ [kcal mol^−1^]	*K*_conf_	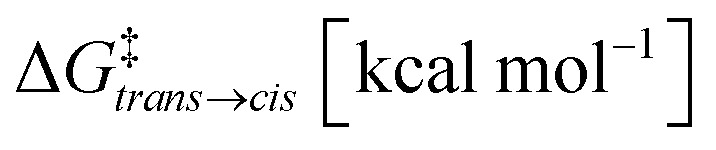	*t*_*trans*→*cis*_ [s]	*t*_*cis*→*trans*_ [s]
2_*trans*_ ⇄2_*cis*_	6.5	1.8 × 10^−5^	22.3	3.5 × 10^+3^	1.4 × 10^−1^
2_*trans*_ ⊂ 1 ⇄ 2_*cis*_ ⊂ 1	5.0	2.3 × 10^−4^	22.0	2.3 × 10^+3^	1.6 × 10^0^
2_*trans*_·2_*trans*_ ⊂ 1 ⇄ 2_*cis*_·2_*trans*_ ⊂ 1^a^	4.0	1.2 × 10^−3^	25.9	1.5 × 10^+6^	4.7 × 10^+2^
2_*trans*_·3 ⊂ 1 ⇄ 2_*cis*_·3 ⊂ 1	2.7	1.1 × 10^−2^	19.8	5.4 × 10^+1^	1.4 × 10^0^

a The second 2_*trans*_ guest was kept in *trans* conformation during the simulation.

The results shown in Fig. 3b compare four cases where: (i) 2 is free in solution (Fig. 3d: dotted curve, *cis* conformer in pink), (ii) 2 is encapsulated in cage 1 (dashed curve, *cis* in dark pink), (iii) the isomerizing 2 is co-encapsulated in cage 1 with another *trans*2 guest (encapsulated 2 dimer: dot-dashed curve, *cis* in light red), and (iv) 2 is co-encapsulated in cage 1 with co-guest 3 (dot-dashed curve, *cis* in light red). The results show that the stability of the conformers of 2 is significantly affected by confinement. The free energy differences between the *cis* and *trans* conformers (Fig. 3b) indicate that, while *trans* is always the most stable configuration of the guest, the *cis* conformer is more and more stabilized as the crowding in the cage cavity increases. The transition barrier also decreases while increasing the crowding. This is captured by the Δ*G* and the *K*_conf_ values, as well as by the relative probability profiles *P*^conf^_*ω*_ shown in Fig. 3b. In particular, the *P*^conf^_*ω*_ is used to plot the relative probability for different conformers (*ω*) of 2 with respect to the *trans* conformer in all simulated complexes. We move from a *cis* : *trans* ratio of ∼10^−7^ : 1 for one 2 free in solution to ∼10^−5^ : 1 in the mono-encapsulated system (2 ⊂ 1), ∼10^−4^ : 1 when two 2 guests are co-encapsulated in the cage (2_2_ ⊂ 1), to ∼10^−2^ : 1 in the 2·3 ⊂ 1 system. The 2 dimer encapsulation (2_2_ ⊂ 1) system, where one of the two guests is kept fixed in a *trans* configuration in accordance with experiments,^10^ falls in between the 2 ⊂ 1 and 2·3 ⊂ 1 cases. The strongly twisted conformation at *ω* = π/4 remains extremely unlikely in all systems, despite a similar thousand-fold stabilization *via* confinement. The most crowded case, 2·3 ⊂ 1, shows a ∼10 000× increase in the probability of finding the more reactive *cis* conformer with respect to the case where 2 is free in solution. This is remarkable, considering that experimentally this case is the one showing the strongest acceleration in the hydrolysis reaction.^10^ These simulations have allowed us to identify the confinement in the cage cavity as a key factor in determining the relative probabilities of the different conformers of guest 2. As a next step, we investigated the propensity of the accessible guest conformers to react.

The encapsulation of amide 2 within cage 1 was shown to enhance amide hydrolysis in a considerable way under mildly basic conditions (experimental results shown in Fig. 1b were obtained at [NaOH] = 100 mM (ref. 10)). To obtain information on the effect of confinement on the hydrolysis reaction (slow-down, inhibition, acceleration, *etc.*), here we modeled the hydrolysis reaction when the amide is free in solution *vs.* when it is encapsulated in the cavity of cage 1. Under moderate pH conditions (6 < pH < 13) and in the absence of other catalysts, the hydrolysis of amides occurs *via* hydroxide attack, forming a tetrahedral intermediate (TI), followed by a second step consisting of the C–N bond rupture.^34^ The first step of the reaction is typically considered the rate determining step of the process^41^ (although in some cases the subsequent bond rupture can contribute to, and also control, the rate of hydrolysis).^34^ For this reason, for the study of the hydrolysis of amide 2, here we focused our investigation only on the formation of the TI *via* nucleophilic attack by a solvated hydroxide (Fig. 3c).

Previously used, *e.g.*, to investigate the hydrolysis of formamide under basic conditions,^41^ here we relied on *ab initio* well-tempered metadynamics (WT-MetaD) simulations^44^ to study the reactivity of 2 free in solution *vs.* confined in the cage cavity. Considering the computational cost of these simulations and the complexity of our systems, we employed a semi-empirical density-functional tight-binding (DF-TB) method,^45,46^ in its self-consistent charge corrected variant SCC-DFTB.^47^ Recently shown to provide comparable accuracy to DFT with large basis sets in terms of prediction of barrier heights and reaction energies for organic molecules,^48–50^ SCC-DFTB has indeed been already successfully used to investigate hydrolysis reactions in biological systems.^51,52^ Despite its approximations, this method guarantees satisfactory accuracy in our case at an affordable computational cost,^24^ a point particularly relevant given that our method of choice, *i.e.* infrequent WT-MetaD, requires running multiple replica simulations in parallel. Noteworthily, SCC-DFTB has been shown to be more accurate than other semi-empirical approaches, *e.g.* AM1 and PM3, for biological applications at a similar computational cost (so that it is now the reference method for, *e.g.*, hybrid QM/MM reaction studies).^53,54^ We simplified our models by studying a system with an OH^−^ near the amide (Fig. 3a), and constraining the C–N–C(O)–C dihedral (*ω*) of amide 2 to representative values, in order to simulate attack on different conformers. We compared 2 conformers with *ω* equal to 0 (*cis* conformer), π (*trans* conformer), and π/4 (a twisted *cis* conformer). We employed replica infrequent *ab initio* WT-MetaD simulations^27^ to obtain information on the reaction coefficients (rate of hydroxide attack and TI formation) for the various conformers of 2 (see the Methods section in the ESI† for details). The reaction barrier of *ω* = π/2 has also been tested, but this conformer was found to be too unstable to compute meaningful kinetic data. From multiple infrequent WT-MetaD runs activating/biasing the transition (R → TI and the TI → R processes), we reconstructed the unbiased kinetics for the transition events and could estimate the characteristic transition times, *τ*_off_ and *τ*_on_. The kinetic constant for hydroxyde release can be calculated as *k*_off_ = 1/*τ*_off_. The kinetic constant for hydroxyde attack (*k*_on_) can be obtained in a similar way from the *τ*_on_ accounting for the OH^−^ concentration in the system. In our simplified setup, the constraint on the OH^−^ is such that the OH^−^ starts in close proximity to the amide (*i.e.*, at solvation distance) and it is not allowed to leave and jump into solution (and to have one water molecule replace its generated void). This is consistent with having an entire solution of OH^−^ ions and having an ion always at a solvation distance from the amide. In other words, this is like having a solvation box where all water molecules are replaced by OH^−^ ions. Such an approximation essentially removes the diffusion of the ion at the reaction site from the study of the reactivity. This is sensible for our purpose, as such diffusion, *i.e.*, the probability of the ion reaching the amide from bulk solution, is directly dependent on the ion concentration, *i.e.*, on the pH of the solution. In terms of the probability of finding an OH^−^ close to the amide, such a constraint effectively produces an effect consistent with having an OH^−^ concentration equal to that of water molecules in pure water (concentration of 55.6 M). Thus, the approximation introduced by the constraint on the amide-OH^−^ distance, in our case is traduced into fixing an OH^−^ concentration of 55.6 M in the system (or of perfectly basic pH = 14).

This simulation approach allowed us to obtain thermodynamic and dynamic characterization of the reaction as a function of the amide conformation (for some relevant discrete *ω* values). The results in Fig. 3d show a strong dependency of reactivity on the *ω* dihedral. As expected, the lowest reactivity against hydroxyde attack was observed for the *trans* conformer, while the *cis* and the twisted *cis* amide conformers were found to be more reactive. In particular, the latter is the only conformer with a *K*_reac_ = *k*_on_/*k*_off_ >1 (Fig. 3d: R is higher in free energy than TI – see complete data in Table S2 in the ESI†). In particular, from the *K* data, we can obtain relative reactivity scores (*χ*_*ω*_) useful to compare the reactivity between the different amide conformers. The *χ*_*ω*_ scores in Fig. 3d (right secondary *y* axis) clearly show how, compared to the twisted *cis* amide conformer (*ω* = π/4), the *cis* amide (*ω* = 0) is ∼1000 times less reactive, while the reactivity of the *trans* amide conformer (π) is basically negligible (∼10^9^ times less reactive than the π/4 conformer).

Altogether, the results shown in Fig. 3 provide a new perspective to obtain a reactivity ranking of the conformers in different systems, which accounts for the configurations of the amide guests which are more/less probable in or out of the cage cavity and their relative reactivity. (i) The most probable conformer in all states, 2_*trans*_, is also the least reactive. (ii) The most reactive twisted conformers (*ω* = π/4 or for *e.g.*, π/2) are, at the same time, highly improbable, even with increased molecular crowding. (iii) The 2_*cis*_ conformer, moderately reactive (but sensibly more reactive than the 2_*trans*_ one), is unfavored in solution against 2_*trans*_, but it becomes more and more relatively favored as the crowding increases upon confinement, emerging as the prominent reactive species in the cage. Our results then converge on the idea that the reaction acceleration by confinement is strongly governed by other dynamic processes of the system, particularly the stabilizing effect of confinement on the more reactive conformers.

The available experimental X-ray structures for these complexes show a 2_*cis*_–2_*trans*_ dimer in the 2_2_ ⊂ 1 case and a 2_*cis*_-twisted conformation in the 2·3 ⊂ 1 complex. This seems to indicate that in these complexes a 2_*cis*_ conformer is more favored than 2_*trans*_. While this may seem to contradict the simulation results discussed above, it is worth noting that all the results collected up to this phase are valid only under the assumption that the encapsulated guests always remain within the cage cavity. Nonetheless, these are host–guest systems, in which the probability of finding the guests within the cage obeys a well-defined supramolecular equilibrium. Estimating the effective probability of finding the guests within the cage requires also studying the dynamics of guest encapsulation/exchange in-and-out of the cavity. As it will be demonstrated in the next section, accounting also for the intrinsic supramolecular dynamics of these host–guest systems provides results that are globally in very good agreement with all available experimental pieces of evidence.

### 2.3 Amide encapsulation/expulsion in-and-out of the cage cavity

**Fig. 4:**
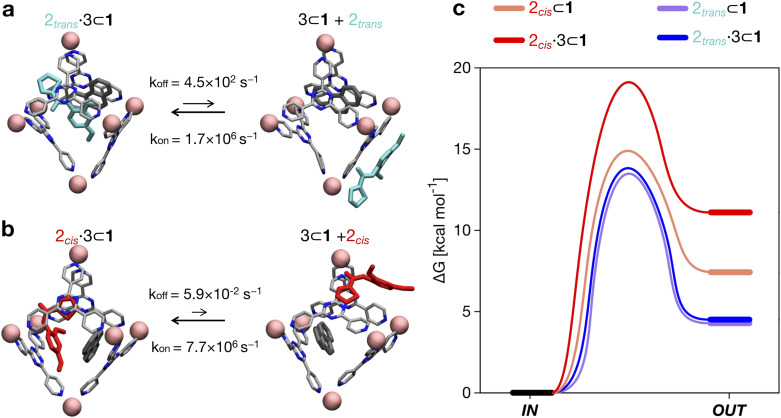
Equilibrium and kinetics of amide guest encapsulation/expulsion in/out of the cage cavity. (a) Equilibrium and kinetics for the encapsulation/expulsion of 2_*trans*_ in/out of the cage when 1 is also hosting guest 3. Above and below the arrows of the equilibrium reaction are respectively the reported kinetic constants *k*_off_ and *k*_on_ estimated from the WT-MetaD simulations. The *k*_on_ values are also reported considering the concentration present in the system (in brackets). (b) Equilibrium and kinetics for the encapsulation/expulsion of 2_*cis*_ in/out of the cage cavity when 1 is also hosting guest 3. (c) Free energy differences and barriers (Δ*G* and Δ*G*^‡^) associated with the encapsulation/expulsion in the cavity of 2 when 1 is also hosting 3 (solid curves) or when 1 does not contain any other guests (dashed curves). Free energy profiles are shown as smoothed fits between the computed critical points.

Up to this point, we have probabilistically compared the reactivity of the amide guest when it is in solution *vs.* when it is encapsulated into the cavity of cage 1. However, to obtain a complete picture, one last point is missing, namely, the effective probability of having such reactive guest conformers in the cage cavity. In fact, such host–guest systems obey a supramolecular equilibrium, where the affinity between the host and the guest dictates the propensity for the guest to stay encapsulated within the cage cavity or to be expelled in solution. Based on our strategy, characterization of the thermodynamics and kinetics of the guest encapsulation/expulsion in-and-out of the cage cavity is thus an additional necessary step. Under realistic conditions, the encapsulation/expulsion of guests as, *e.g.*, 2 or 3 in/out of the cavity of cage 1 may require crossing considerably high free energy barriers,^16,32^ which makes them rare events in the timescales accessible *via* classical atomistic MD simulations. As recently carried out for other host–guest^16^ and dynamic supramolecular systems,^33,55^ we thus reconstructed the thermodynamics and kinetics for the processes of encapsulation/expulsion of amide 2 in/out of the cavity of cage 1 by means of a well-suited WT-MetaD^56^ simulation protocol (complete computational details are available in the ESI†).^16,33,55,57,58^ We underline that, while the possibility to estimate transition kinetics and then barriers from infrequent WT-MetaD simulations rather than from converged (recrossing) WT-MetaD simulations has been reported previously for other systems,^57,58^ including dynamic supramolecular ones,^16,33,55^ given the complexity of the systems studied herein, this stands out as a very efficient, robust and powerful approach. The extracted data are collected and shown in Table 2 and Fig. 4.

**Table 2:** Equilibrium and kinetics of the amide encapsulation/expulsion in/out of the cavity. For each simulated host–guest complex, encapsulation free energies (Δ*G*), equilibrium constants *K*_enc_, expulsion free energy barriers 
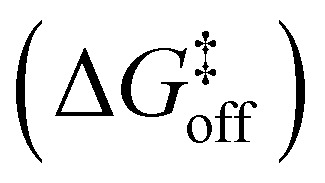
, characteristic in-cavity residence times (*t*_off_), and the associated transition rates (*k*_off_ and *k*_on_) estimated from the WT-MetaD simulations are reported

Process	Δ*G* [kcal mol^−1^]	*K*_enc_	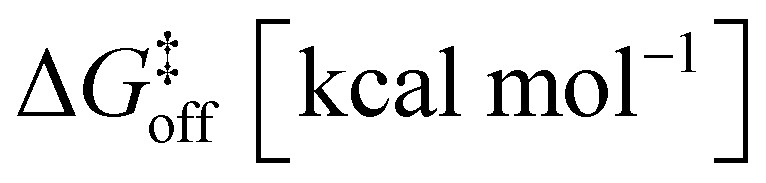	*t*_off_ [s]	*k*_off_ [s^−1^]	*k*_on_ [s^−1^]
2_*trans*_ + 1 ⇄ 2_*trans*_ ⊂ 1	−4.3	1.5 × 10^3^	13.5	1.2 × 10^−3^	8.3 × 10^2^	1.3 × 10^6^
2_*cis*_ + 1 ⇄ 2_*cis*_ ⊂ 1	−7.4	2.1 × 10^5^	14.9	1.4 × 10^−2^	7.1 × 10^1^	1.8 × 10^7^
2_*trans*_ + 2_*trans*_ ⊂ 1 ⇄ 2_*trans*_·2_*trans*_ ⊂ 1	−4.7	2.7 × 10^3^	14.5	7.3 × 10^−3^	1.4 × 10^2^	3.7 × 10^5^
2_*cis*_ + 2_*trans*_ ⊂ 1 ⇄ 2_*trans*_·2_*cis*_ ⊂ 1	−9.3	6.9 × 10^6^	16.7	9.6 × 10^−1^	1.0 × 10^0^	7.1 × 10^6^
2_*trans*_ + 3 ⊂ 1 ⇄ 2_*trans*_·3 ⊂ 1	−4.5	2.2 × 10^3^	13.8	2.2 × 10^−3^	4.5 × 10^2^	1.0 × 10^6^
2_*cis*_ + 3 ⊂ 1 ⇄ 2_*cis*_·3 ⊂ 1	−11.1	1.3 × 10^8^	19.1	1.7 × 10^1^	5.9 × 10^−2^	7.7 × 10^6^

By comparing the encapsulation of the different isomers of 2 in the cavity of cage 1, either alone or when 1 also contains a co-guest (3 or 2_*trans*_), we could observe that in general the encapsulation of 2_*cis*_ is more favored than that of the 2_*trans*_ isomer in all studied cases (see Table 2 and Fig. 4). The kinetic constants measured for the 2_*trans*_ encapsulation/expulsion in/out of the cavity indicate that in all complexes the dynamics of the transitions are marginally affected by the presence of other guests in the cage (*k*_off_ and *k*_on_ in the same orders of magnitude). On the other hand, the dynamics (and stability) of the 2_*cis*_-complexes is more impacted by the presence of co-guests in the cage cavity, which are found to stabilize the 2 encapsulation in the cage cavity by ∼2–4 orders of magnitude in the presence of 3 (lower *k*_off_: more improbable/slower 2 expulsion out of the cavity). As shown in Table 2, the estimated *k*_off_ for the expulsion of the 2_*cis*_ guest out of the cage cavity drops from ∼7.1 × 10^1^ s^−1^, when only one 2_*cis*_ is present in the cavity of 1, to ∼1 s^−1^ or ∼5.9 × 10^−2^ s^−1^, when 2_*cis*_ is co-encapsulated in the cavity of 1 with a 2_*trans*_ or with a 3 co-guest respectively. The *k*_on_, on the other hand, is found to be globally similar in all simulated cases (see Table 2).

Altogether, these data suggest that differences in the host–guest equilibrium in such systems are mainly controlled by the interactions/affinity between the guest (2) and the effective host cavity, thought of as that accessible for the guest considering the cage and the presence of eventual encapsulated co-guests. From the calculated Δ*G*, we could also estimate the host–guest affinity constants (*K*_enc_) for all the host–guest systems (Table 2). It is worth noting that the *K*_enc_ values for the 2_*cis*_ conformers are in general orders of magnitude higher than those of the 2_*trans*_ complexes. In particular, this is evident for the 2·3 ⊂ 1 complexes. In such a case there is a difference in the encapsulation *K* of ∼5 orders of magnitude (Table 2). This means that the probability of having 2_*cis*_ co-encapsulated in the cavity together with 3 is ∼100 000× higher than that of finding a co-encapsulated 2_*trans*_. Despite the fact that, in theory, the 2_*trans*_ conformer is found ∼100× more favored compared to the 2_*cis*_ one in the cage cavity (see Fig. 3d: right secondary axis), such a statistical penalty for having a 2_*trans*_ conformer effectively encapsulated within the cage cavity – emerging from the host–guest equilibrium – explains why the experimentally obtained X-ray structures of these complexes always show a 2_*cis*_ encapsulated guest.

Nonetheless, the high *K*_enc_ values indicate that in all the cases simulated herein the amide guest can be, in good approximation, assumed as always encapsulated in the cage cavity. In fact, from the thermodynamic data we can extrapolate the partition probability *P*^in^ = *K*_enc_/(1 + *K*_enc_). *P*^in^ indicates the relative probability of having 2 in *vs.* out of the cage cavity. In particular, *P*^in^ tends to be 1 when the *K* is high and the guest has a high probability of being encapsulated in the system, as it is the case in general for all host–guest complexes explored herein (*P*^in^ ∼ 1 in all cases, see also the next section).

### 2.4 Molecular determinants of reactivity in dynamic host–guest systems

**Fig. 5:**
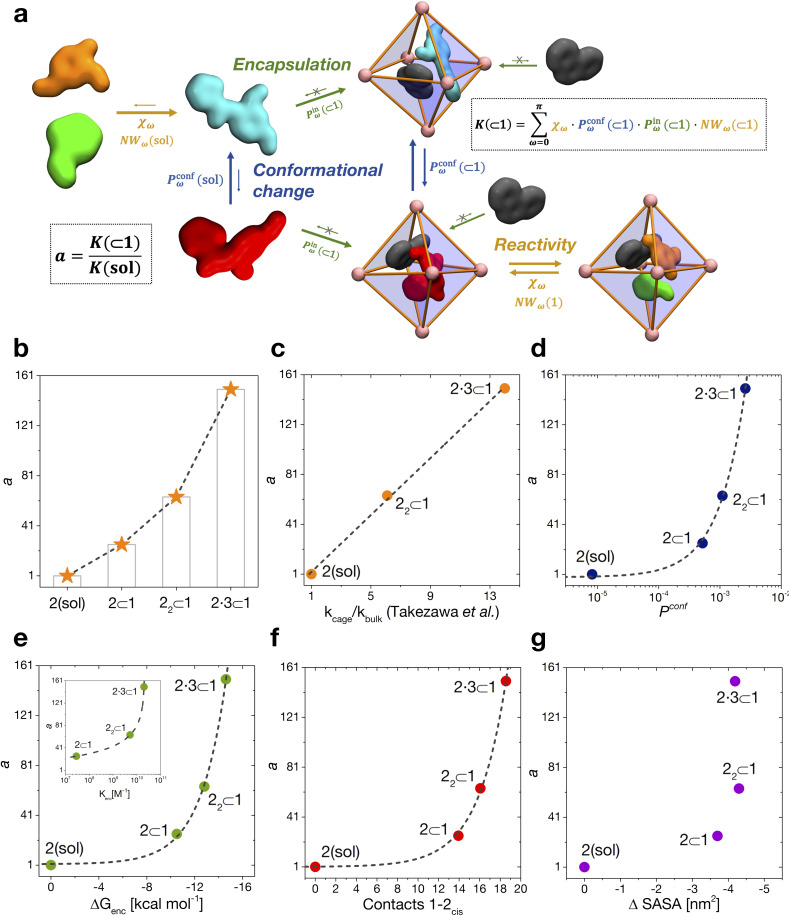
Reaction acceleration in a dynamic host–guest system. (a) Full dynamic representation scheme of the investigated host–guest system, showing the processes that need to be taken into account to rationalize the reaction acceleration observed experimentally. (b) Computed reaction acceleration for the various investigated host–guest systems: the hydrolysis acceleration index, *a*, is expressed relative to reactivity of the 2 guest alone in solution (see eqn (1)–(6)). (c) Correlation between the reaction acceleration, *a*, computed from the simulations and the acceleration measured experimentally^10^ (linear fit reported by the dashed line). (d–h) Relationships between the computed reaction acceleration *a* and various characteristic parameters of the host–guest systems: (d) relative probability of finding the 2_*cis*_ conformer over the 2_*trans*_ conformer in solution *vs.* in the different host–guest complexes (dashed line: exponential fit); (e) encapsulation free energy Δ*G*_enc_ of the 2_*cis*_ conformer in the different complexes (exponential fit reported by the dashed line); (f) the weighted number of contacts between the host and the guest, evaluated as the product of the peak position of the distribution shown in Fig. 2c and its height; (g) the reduction in the solvent accessible surface area (SASA) of the 2_*cis*_ guest in the different encapsulation complexes *vs.* when this is in solution (an indirect measure of the solvophobic effect,^55,59^ showing no clear correlation).

We have thus far investigated, separately, the three main processes that concur to define the global dynamic picture of amide hydrolysis under confinement (Fig. 5a): (i) encapsulation/expulsion of the guests in-and-out of the cage cavity; (ii) isomerization around the *ω* dihedral in solution and in confinement; (iii) the reaction itself. For each of them, we have devised a probability metric and observed how it varies across the different investigated cases. Our last step is thus to put these processes (and probabilities) together, in order to capture the full dynamic complexity of the system.

To this end, we can define a reaction acceleration index, *a*, as the ratio between the observed reactivity with or without the presence of a cage in the system – *i.e.*, when the reactant, guest 2, is encapsulated within the cage cavity (*K*( ⊂ 1)) or when it is free in solution (*K*(sol)):

1 *a* = *K*(⊂ 1)/*K*(sol)

In the real system, amide hydrolysis can in principle take place both when 2 is encapsulated in the cage cavity and when it is out of the cage (with the observed reaction coefficients determined by the probabilities for finding the different reactive conformers – planar or twisted – in the two environments). In general, the reaction acceleration *a* will thus depend on the likelihood that the hydrolysis of 2 occurs in *vs.* out of the cage. From our simulations we have seen that the conformational free energy landscape of the amide guest may change upon encapsulation (changing the relative free energy difference between *cis* and *trans* conformers). As a consequence, the probability of crossing the rotational barrier around the amide bond also changes. In particular, we could observe that the more reactive 2_*cis*_ conformer is more and more stabilized as the crowding increases in the cavity of cage 1 (Fig. 3d). The simulations also show that the encapsulation of 2_*cis*_ within the cage cavity is considerably more stable than that of 2_*trans*_, showing a higher affinity and retention time (Fig. 4 and Table 2). Altogether, this indicates that it is more likely to observe 2_*cis*_ rather than 2_*trans*_ encapsulated within the cavity of the cage, which is consistent with the fact that the 2_*cis*_ conformer is present in the crystal structures obtained experimentally.^10^

The reactivity in the system depends on the propensity of the visited 2 conformers to react, their relative population in the different complexes, their probability of encapsulation (*i.e.*, the relative population ratio between having 2 in the cage *vs.* in solution at the equilibrium), and the solvent molecule accessibility to the amide (*i.e.*, the solvent is another key reactant) upon encapsulation. Noteworthily, all these parameters can be extracted from our simulations.

In general, we can define a global reaction constant for the case when hydrolysis takes place within the cage cavity, *K*(⊂ 1), as the sum of the reaction constants (*K*_*ω*_(⊂ 1)) for all amide conformers (*ω*) visited by the guest reactant 2 in the cage cavity:2
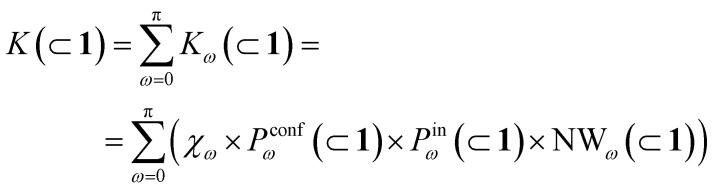
where *χ*_*ω*_ is the hydrolysis reaction constant associated with the possible amide conformers *ω* (see Fig. 3d), *P*^conf^_*ω*_(⊂ 1) is the relative statistical weight for all different conformers *ω* in the cage cavity (Fig. 3b), *P*^in^_*ω*_(⊂ 1) is the probability of effectively having each specific conformer *ω* in the reactive environment – in this case, inside the cage cavity (see Table 2) –, and NW_*ω*_(⊂ 1) is the average number of contacts between solvent molecules (water or OH^−^: key reactants for amide hydrolysis) and the amide's carbonyl (estimated as shown in Fig. 2e).

Accordingly, the global reaction constant in the absence of the cage in the system (2 alone in solution), *K*(sol), can be defined as:3
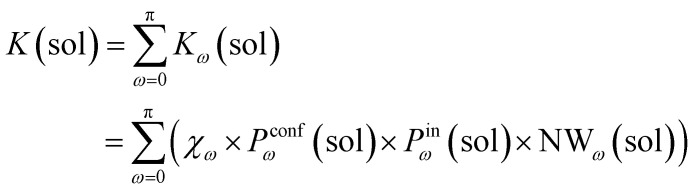
where in this case *P*^conf^_*ω*_(sol) and NW_*ω*_(sol) refer respectively to the relative probabilities for 2, when alone in the solvent, to assume the different conformers *ω*, and the corresponding number of amide carbonyl-solvent molecules contacts. In this case, in the absence of the cage in the system, guest 2 is by definition always out of the cage, and *P*^in^_*ω*_(sol) = 1. Thus, eqn (3) is simplified into:4
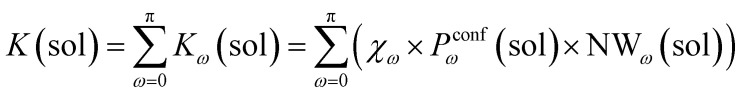


Transforming the formula for acceleration into:5
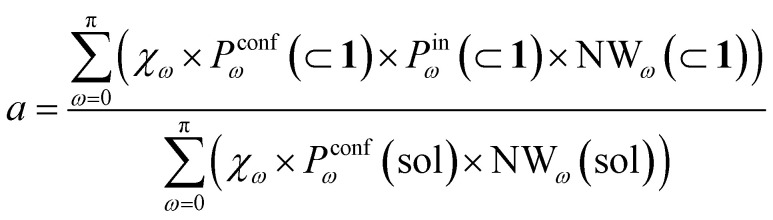


Moreover, it is worth noting that, given the high Δ*G* values in Fig. 4c and Table 2, when the cage is present in the system, the guests can also be considered to be always encapsulated within the cage cavity, so that in eqn (2) the *P*^in^(⊂ 1) term tends to be ∼1 (*vide supra*):6
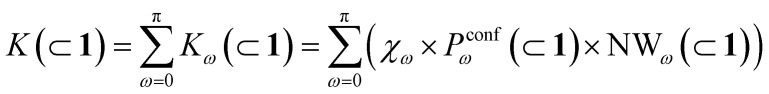


Reducing the formula for the acceleration to:7
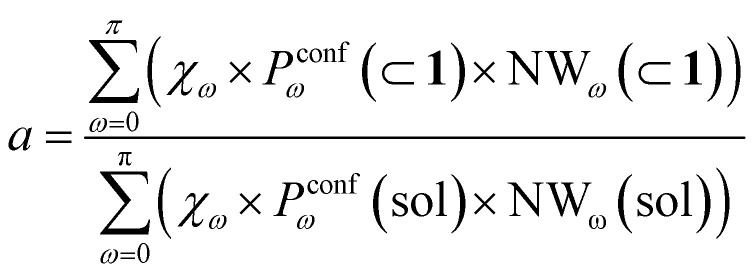


This means that, given the high propensity for guest encapsulation (*P*^in^(⊂ 1) ∼ 1), in this specific case the reaction acceleration in the system is found to be little dependent on the guest encapsulation/expulsion equilibrium. On the other hand, the reactivity turns out to be rather controlled by the fact that the guest is more favored to assume reactive conformations inside the cage cavity (compared to the case when this is free in solution). This is in full agreement with the available experimental evidence on these systems.^10^

Finally, it is worth noting that while the summations in eqn (6) and (4) run in principle over all possible values of *ω* (have different reactivities – see Fig. 3b), the data of Fig. 3d clearly show that, due to the intrinsically high isomerization barrier, the relative probability of observing twisted (an extremely reactive) 2 conformers (*e.g.*, π/2, π/4, *etc.*) is very low. These are distorted, very unstable conformers, with a survival lifetime which tends to be 0, and for which the product *χ*_*ω*_*P*^conf^_*ω*_ ∼ 0. The unique conformers with survival life and *P*^conf^_*ω*_ ≠ 0 are 2_*cis*_ (*ω* = 0) and 2_*trans*_ (*ω* = π). The latter, however, is substantially non-reactive (Fig. 3b: *χ*_π_ ∼ 0), so that also in this case *χ*_π_*P*^conf^_π_ ∼ 0. Based on these observations, in our case the reactivity of the system seems thus to be largely related to (i) how much over-stabilized the reactive 2_*cis*_ conformer is and (ii) how accessible the amide is to the co-reactant solvent molecules in such conformation in the cage cavity *vs.* in solution.

By combining these data, we estimate the reaction acceleration *a* for the various host–guest complexes reported in Fig. 5b. We observe that, in this case, the reactivity increases with the crowding in the system. While a ∼26-fold acceleration is computed for the mono-encapsulated case (2 ⊂ 1), a double-encapsulation gives a ∼64-fold increase for the 2_2_ ⊂ 1 system. A dramatic *a* ∼ 150 is obtained for the 2·3 ⊂ 1 complex. While such estimated *a* values may differ quantitatively from those obtained from the experiments (this can be expected, given the deviations of such ideal models from realistic systems/conditions), the trends can still be safely compared. Fig. 5c shows a remarkable trend between our calculated acceleration data and the experimental ones. This validates our simulation approach. It is worth noting that the mono-encapsulation case (2 ⊂ 1) does not have an experimental counterpart, due to the tendency of 2 to dimerize within the cage. Nonetheless, this extra-case (where crowding is lower than that in, *e.g.*, 2_2_ ⊂ 1 and 2·3 ⊂ 1) provides an additional case useful for comparison. In particular, the limited computed acceleration seen in this case supports the evidence that molecular crowding within the cage cavity is a key player in the reactivity in the host–guest system.

In order to obtain an insight into the key molecular determinants controlling the reaction acceleration in these host–guest systems, as shown in Fig. 5d–g we plot the computed *a* parameters against some of their key constitutive terms.

We have seen that the difference in affinity between *cis* and *trans* conformers among the different systems is the main factor determining the final reaction acceleration using eqn (1), this being shown by the nearly perfect exponential correlation between *a* and the relative probability of finding the 2_*cis*_ conformer with respect to the 2_*trans*_ conformer in solution and in the different encapsulation complexes (Fig. 5d). The trend suggests that small incremental stabilizing effects on this conformation, *e.g.* by changing affinity and size of the co-guest, could result in potentially outstanding enhancements of reactivity for guest 2, keeping all other parameters constant.

Noteworthily, a quasi-exponential trend is observed between the computed reaction acceleration *a* and the encapsulation free energies (Δ*G*_enc_) for 2_*cis*_ in all systems (Fig. 5e). In these systems, where the reactivity is observed to increase with the crowding inside the cage cavity, *a* is also clearly related to the host–guest interaction (namely, to obtain a stable complexation, a strong host–guest affinity is necessary to compensate for the crowding penalty associated with the binding). This affinity, as evidenced by a qualitative investigation of how the different components interact with each other, both in our simulations and in the available experimental X-ray structures,^10^ is mainly driven by π–π stacking between the four triazine ligands of cage 1 and the aromatic rings of amide 2, particularly favorable in the *cis* conformation and by residual solvophobic interactions between such ring groups. A similar trend can also be observed when looking at the weighted number of contacts between the host and the guest (Fig. 5f, evaluated from the distributions shown in Fig. 2c, *i.e.* a proxy for the host–guest interaction energy). If we consider the interaction between 2 and cage 1 to be consistent among all the investigated systems, we can trace the trend back to the interaction between the guest and co-guest (or the absence thereof in the 2 ⊂ 1 case), with 3 showing a greater stabilizing effect for 2_*cis*_ within the cage cavity compared to another 2 co-guest. Noteworthily, as revealed by the obtained trends shown in Fig. 5e and f, such favorable affinity can stabilize the reactive conformer of amide 2 to a higher extent, which results in a remarkable increase in the reaction acceleration *a*. The remarkable stabilizing effect of co-guest 3 is driven by a balance between having an aromatic structure able to participate in π–π stacking with guest 2, its considerable solvophobicity that makes its encapsulation within the cage cavity very stable, and a size (volume = 172 Å^3^)^39^ that allows the amide to reside comfortably in the cavity in a *cis* (reactive) conformation. Another 2 co-guest, while still being able to engage in favorable interactions (Table 2: host–guest complexation on average more favorable than that of a single 2), has a larger volume (241 Å^3^)^39^ which constrains the first guest in less favorable configurations and produces a less stable complex.

To obtain information on how much of the host–guest interaction is due to solvophobic effects, we calculated the reduction in the solvent accessible surface area (SASA) of the 2_*cis*_ conformer when it is encapsulated in the cavity of the various complexes *vs.* when it is alone in solution.^16,55,59^ While a correlation with the computed reaction acceleration is observed (see Fig. 5g), the trend becomes less neat. The trend is respected while moving from the amide in solution to the mono-guest (2 ⊂ 1) and double-guest complexes (2_2_ ⊂ 1 and 2·3 ⊂ 1). However, the differences in acceleration between the various systems do not correlate in a neat manner with the ΔSASA calculated for the various cases. This reveals that (i) non-specific hydrophobic effects alone are not sufficient to grasp the complexity of these reactive systems and suggests that (ii) like in most receptor-ligand complexes in nature, specific molecular interactions are probably relevant in controlling host–guest affinity.

## 3 Conclusions

Understanding reactivity in dynamic regimes and in systems in which reactants and products are in continuous exchange is a non-trivial task. Here we report a computational approach that allows us to reconstruct the reactivity in dynamic host–guest systems and to study in atomistic detail the key (molecular and dynamic) factors that control it. As a case study, we focus on the hydrolysis of amide guests encapsulated in the cavity of a coordination cage, for which experimental evidence for reaction acceleration has been recently reported.^10^ By combining a multi scale modeling scheme with metadynamics simulations, we couple the study of the intrinsic dynamics of the host–guest system with that of the amide hydrolysis reaction. The approach allows us (i) to characterize the barriers to the hydrolysis reaction as a function of the conformation assumed by the amide, (ii) to study the conformations that the amide guest can assume and estimate their relative probabilities in *vs.* out of the cage cavity, and (iii) to characterize the dynamic processes of guest encapsulation/expulsion in/out of the cage. Altogether, we demonstrate how this allows us to reconstruct the reactivity in such dynamic host–guest systems by calculating the relative reactivity of the various molecular conformers/arrangements that the amide guest can assume and to what extent the reactive conformations are more/less favored in *vs.* out of the cage, by re-weighting the results considering the probability of effectively having the guest encapsulated inside the cage cavity. This allows us to estimate a reaction acceleration score, which estimates to what extent the reaction is accelerated/hindered in the host–guest system and allows comparing between system variants.

In the specific case study used here, we obtain clear evidence that the reaction acceleration is controlled by the crowding effects accompanying the guest encapsulation. We compared four cases, where the amide guest 2 is alone in solution, when it is encapsulated in the cage (2 ⊂ 1), or when it is co-encapsulated together with other co-guests in the cage cavity (2_2_ ⊂ 1 and 2·3 ⊂ 1). We characterized all of them, and we estimated their hydrolysis acceleration factor. The results show unambiguously that the encapsulation of the amide in the cage cavity tends to stabilize the reactive conformers of the amide guest. This is key in such specific systems, considering the high encapsulation constants obtained for all studied cases (*i.e.*, high probability of finding the amide guest within the cage cavity). We clearly observe how, when a co-guest is also co-encapsulated with the amide guest in the cage, the crowding in the cavity augments and the reactive *cis* conformer of the amide guest is more and more stabilized. The acceleration scores estimated from our computations are found to have remarkable trends with the reaction acceleration observed experimentally. Overall, our computational results are found in optimal agreement with the experimental results by Takezawa *et al.*,^10^ while it is worth noting that in our approach these emerge bottom-up, from a comprehensive study of the molecular and supramolecular dynamics of the host–guest system and of its key molecular equilibria. This provides a general character to this approach, as confinement-induced reaction acceleration (or deceleration) in such supramolecular (and intrinsically dynamic) host–guest systems can only be explained by taking into account all the dynamic processes that occur within them. The comprehensive picture of hydrolysis and of how this may be modulated under confinement that we obtain here provides a general high-resolution framework for building structure–property relationships. This approach constitutes a useful general-purpose platform, useful to explore strategies towards the rational design of host–guest systems with a molecular-level control of chemical reactivity. Based on the results that we show herein, this approach offers a useful platform to explore, *e.g.*, the effect of tuning the properties/features of the guest (*e.g.*, hydrophobicity, symmetry, interactions, size, *etc.*) or of the cage cavity (*e.g.*, structure, flexibility, hydrophobicity, *etc.*), as recently shown in other host–guest reactive systems.^16^ Its versatility and generality may also be useful to explore ways to gain control over system reactivity by tuning host–guest dynamics by using, *e.g.*, guest mixing and guest–guest encapsulation competitions,^10^ which could allow tuning the residence time and fractions of encapsulated guests within the cage cavity and, consequently, the reaction in the system. The approach described herein appears to be well suited for the case studied, and it is general enough that it can be useful to also study other types of dynamically reactive systems. While very effective in our case, the reconstruction of the dynamic reactivity by correcting this with weights that account for effective (probabilistic) reactant availability and their propensity to react has some intrinsic limitations. For example, in our case it does not explicitly consider the possible active roles of intermediate states that can promote/hinder the reactivity. Also, in this specific case in the calculations of reactivity we do not explicitly account for the effect of the cage on the hydrolysis reaction. In our case, the effect of the cage is accounted for implicitly: this enters into play as the encapsulation of the guest within the cavity favors reactive amide conformers or it allows for fewer solvent molecules (catalyst) to reach the proximity of the amide compared to that when the amide is in solution. Clearly, this is a valid assumption as long as the structure of the cage does not have a catalytic role in the reaction (as it is assumed to be the case in such systems).^10^ Nonetheless, it is worth noting that the versatility of our approach makes it well suited for methodological extensions. For instance, when needed, it is in principle possible to include explicitly the cage in the study *via*, *e.g.*, a hybrid QM/MM approach where the reactive sites are modeled explicitly at the quantum level while the surroundings are treated with classical atomistic force fields. Another methodological limit is related to the complexity of the reaction and of the system as a whole. One practical limit of MetaD simulations lies, in fact, in the possibility to choose a reduced number of appropriate collective variables that can effectively and correctly model all the phases from (ii) to (iv) of this study. While in this case MetaD simulations are effective, it is worth noting that the approach must not necessarily be built on MetaD simulations. Any enhanced sampling method that allows the probabilistic factors of eqn (2) and (3) to be reliably estimated would be equally effective. In this sense, the fact that the workflow presented herein is built on a concept (*i.e.*, combining the study of reactivity with that of the intrinsic dynamics of the system) but not on a specific method provides flexibility and robustness to the approach. Furthermore, the results we report herein demonstrate how, despite the relatively simple concepts on which our approach is built, this is effective, and it offers concrete opportunities to reconstruct reactivity in dynamical host–guest supramolecular systems. It is also worth underlining how, in achieving such goals, this approach offers a first attempt to link the typical computational chemistry study of chemical reactions to that of a complex dynamic reaction process, more typical of chemical engineering.

In general, all these considerations underline how gaining control over the dynamics of these systems is key to controlling reactivity within them and indicate how approaches such as those described herein can offer relevant support towards the development of new types of reactive supramolecular systems.

## Data availability

Complete computational materials and data pertaining to the study conducted herein are available at: https://doi.org/10.5281/zenodo.7064016.

## Author contributions

M. D. P., L. P. and M. C. created the molecular models and performed the simulations. All authors contributed to the analysis of the results and to the writing of the manuscript. G. M. P. conceived the research and supervised the work.

## Conflicts of interest

There are no conflicts to declare.

## Supplementary Material

SC-013-D2SC02000A-s001
