# Supplementary material for: Reconstructing reactivity in dynamic host–guest systems at atomistic resolution: amide hydrolysis under confinement in the cavity of a coordination cage
Source: Chem Sci. 2022 Aug 29;13(37):11232–45. doi: 10.1039/d2sc02000a (PMC9517058; doi:10.1039/d2sc02000a)
Supplement: SC-013-D2SC02000A-s001 [file SC-013-D2SC02000A-s001.pdf]

Electronic Supplementary Material (ESI) for Chemical Science.
This journal is © The Royal Society of Chemistry 2022

# Supplementary Information for:

# Reconstructing Reactivity in Dynamic Host-Guest Systems at Atomistic Resolution: Amide Hydrolysis Under Confinement in the Cavity of a Coordination Cage

Massimo Delle Piane,[†] Luca Pesce,[‡] Matteo Cioni,[†] and Giovanni M. Pavan[*,†,‡]

†*Department of Applied Science and Technology, Politecnico di Torino, Corso Duca degli Abruzzi 24, 10129 Torino, Italy*

‡*Department of Innovative Technologies, University of Applied Sciences and Arts of Southern Switzerland, Polo Universitario Lugano, Campus Est, Via la Santa 1, 6962 Lugano-Viganello, Switzerland*

**E-mail: giovanni.pavan@polito.it**

# Computational methods and supplementary figures

## Creation and parametrization of the molecular models

All atomistic model systems have been parametrized based on General Amber Force Field (GAFF)[1], with the exception of the amide $\omega$ dihedral angle, which has been reparametrized to improve its accuracy as explained in the following section below. The donor-acceptor (Pd-N) bonds were parametrized using Seminario's method[2] following the Metal Center Parameter Builder (MCPB) protocol[3] as recently done in the atomistic models of other coordination cages.[4] The partial charges for the whole atomistic system (cage and the guests) were calculated using the RESP approach[5] as implemented in CP2K.[6] The quantum mechanical calculations for this purpose were performed using CP2K, with a BLYP-D2 functional[7,8], paired with the Goedecker–Teter–Hutter pseudopotentials[9] and a triple-$\zeta$ basis set with polarization functions (TZVP).[10] The atomistic parametrization was then carried out using the ANTECHAMBER software.[11]

All the atomistic simulations in this work have been conducted in explicit solvent using the GROMACS-2020.2 software[12] patched with Plumed-2.7.[13] All systems were immersed in a simulation box filled with explicit TIP3P water molecules.[14] All simulated systems consist of a cubic box of different side length depending on the system: 37.2 Å for the amide in solution solvated by 1684 water TIP3P molecules, and ∼51.0 Å with ∼4290 water molecules for the other 3 host-guest systems. Moreover, for those models containing the cage, 12 molecules of $NO_3^-$ have been also added to neutralize the total charge present in the systems, consistent with the experimental conditions.[15]

## Reparametrization of $\omega$ dihedral potential terms of amide 2

Peptide bonds in protein structures are mainly found in *trans* conformation with a torsion angle $\omega$ close to $\pi$. Due to the small population of the *cis* conformation[16,17], the kinetics of isomerization of peptide bond has always been difficult to characterize experimentally and,

as a consequence, only limited data on the free energy barriers separating the two isomers are available.[18] Being generally derived from quantum mechanical calculations on model compounds, or based on experimental data from thermodynamic and kinetic studies,[19,20] the AMBER force field shows limitations in the estimation of the accurate free energy difference between the *cis* and *trans* conformations in peptide bond. Herein, this was evidenced and proven by preliminary metadynamics tests in this sense. As showed in section **??**, the description of the *cis*-*trans* equilibrium for the amide **2** has a central role in the study of its reactivity, and thereby, our aim is to evaluate the accuracy of the potential terms involved in the process of isomerization of the $\omega$ dihedral angle. In the AMBER force field, the torsional potential energy term is expressed as the Pitzer potential[21], a Fourier series term given by:

$$\sum \frac{V_n}{2}[1 + cos(n\mu - \gamma)] \tag{S1}$$

where $V_n$, n, $\mu$, and $\gamma$ are, respectively, the dihedral force constants, periodicity, torsional angle, and phase angle.

To reproduce the torsional energy profile of the peptide bond, only the first two terms in this series are relevant: $V_1$ (n=1, $\gamma$ = 0), and $V_2$ (n=2, $\gamma$ = 180); the former describes the *cis*-*trans* equilibra, while the latter is responsible for the barriers to rotation about C-N bond.

In order to test the accuracy of the dihedral amide parameters, we compared the free-energy surface (FES) for the torsion of an N-methylacetamide (NMA) amide – the simplest analog of the peptide bond within amides, that here we use as a reference to optimize the amide force field parameters – obtained performing WT-MetaD simulation with the experimental energy profile obtained with NMR studies.[18] In these WT-MetaD simulations, as the collective variable (CV) we chose the $\omega$ dihedral angle of the amide, with a bias factor of 30 deposited every 500 steps using Gaussians of initial height of 1.2 kcal/mol and a $\sigma$ of 0.35 rad. Our simulations show that the default $\omega$ AMBER potential parameters fail to

reproduce correctly the *cis-trans* equilibrium and free energy barriers for the isomerization compared to the experimental estimates, shown in Figure S1 (red *vs.* blue). In order to obtain a proper description of the $\omega$ isomerization in our models, first we replaced the standard AMBER force field values for the dihedral potential with those previously pre-optimized by Doshi and Hamelberg[22], which have been here optimized further in order to improve the agreement with the experimental data (green *vs.* blue).

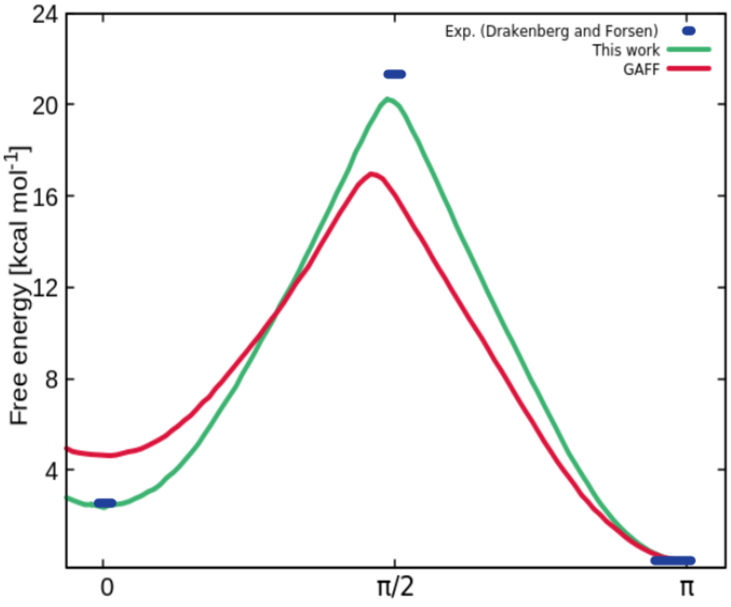


| | $\Delta G^{\ddagger}_{trans \rightarrow cis}$ | $\Delta G^{\ddagger}_{cis \rightarrow trans}$ | $\Delta G_{cis \rightarrow trans}$ |
|---|---|---|---|
| Experimental | 21.3 | 18.8 | 2.5 |
| This work | 20.3 | 18.0 | 2.3 |

Figure S1: **Comparison between the FESs obtained with our reparametrized dihedral potential terms (in green) or with the original GAFF parameters (in red) *vs.* the experiments (blue).**[18]

More precisely, these dihedral force field parameters have been modified until a good agreement was reached between our *cis-trans* isomerization FES and the experimental free energy profile for NMA (see Figure S1), modifying the main torsional parameters involved in $\omega$ bond rotation, while all the other non-bonded parameters were left unchanged (Table S1: in our procedure only the $V_2$ term for the general X-C-N-C torsion, and the $V_1$ and $V_{2'}$ potential parameters specific of the H-N-C-O torsion have been modified).

Table S1: Comparison between the $\omega$ torsional potential parameters in GAFF or in the improved AMBER force field parameters by Doshi and Hamelberg *vs.* those optimized in this work (values are expressed in kcal $mol^{-1}$).

| | $V_n$ | | | |
|---|---|---|---|---|
| Torsion | GAFF | Doshi and Hamelberg | This work | $\gamma$ |
| X-C-N-X | 20.0 | 28.0 | 27.0 | 180° |
| H-N-C-O | 5.0 | 5.8 | 6.8 | 180° |
| H-N-C-O | 2.0 | 1.0 | 1.0 | 0° |

## Classical atomistic molecular dynamics simulations

All simulated systems ($\mathbf{2} \subset \mathbf{1}$, $\mathbf{2}_2 \subset \mathbf{1}$, $\mathbf{2} \cdot \mathbf{3} \subset \mathbf{1}$, with **2** in both *cis* and *trans* conformations) have been preliminarily minimized and then equilibration was performed first in the NVT (constant N: number of particles, V: volume, and T: temperature, during the run) ensemble at 300K and then in the NPT (constant N: number of particles, P: pressure, and T: temperature, during the run) ensemble at 300 K and 1 bar. Production runs were then conduced in NPT simulations for up to 1 $\mu$s of MD. All simulations were conduced in periodic boundary NPT conditions. All simulations used the v-rescale thermostat[23] and the Berendsen barostat[24] to keep a 300 K of temperature and 1 bar of pressure in the systems. The electrostatic interactions have been treated using the Particle Mash Ewald (PME).[25] The cutoff for the real part of the summation was 1.0 nm. The cutoff for the van der Waals interactions was set to 1.0 nm. All of the bonds involving hydrogen atoms were constrained using the LINCS algorithm.[26] The leap-frog integrator was used to integrate the equations of motions. All the analyses of Figure 1 in the main paper have been performed with Plumed-2.7.[13]. For the contact analysis a distance cutoff of 0.5 nm has been chosen between the involved groups, paired with a switching function with parameter $D_0$=0.35 nm. The Solvent Accessible Surface Area (SASA) was computed with the default probe radius of 0.14 nm.

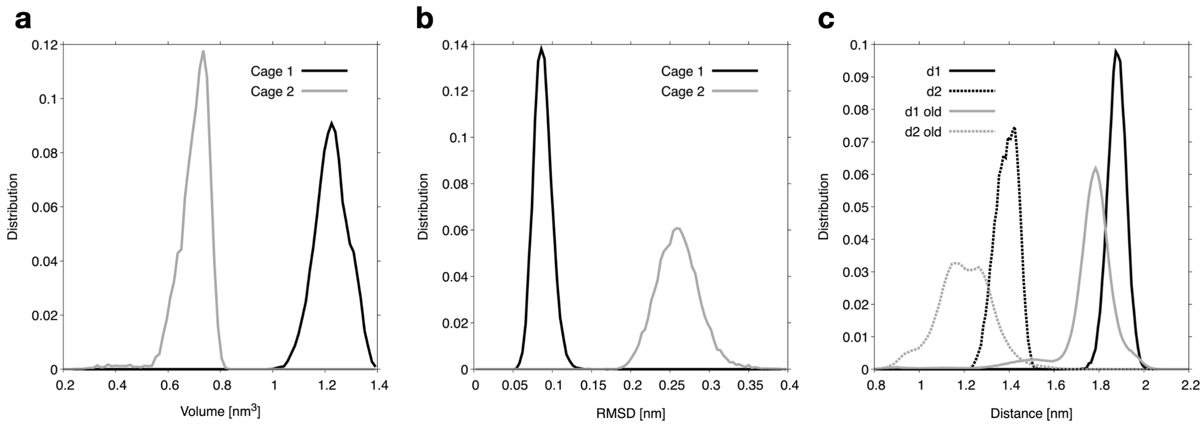


Figure S2: **Conformational landscape of cage 1 compared to that of another coordination cage type**. Cage **1**, investigated in this paper, is very rigid compared to another recently modeled coordination cage (**2**),[27] which was conversely demonstrated to be flexible.[4] (**a**) Histograms of cage cavity volumes. (**b**) Histograms of the Root Mean Square Deviation (RMSD) of the atomic positions of the two cages. (**c**) Histograms of two variables – d1: axial distance between the "top" and "bottom" Pd atoms in the cages, and d2: the distance between the midpoints of the two opposite equatorial edges of the octahedral cages.

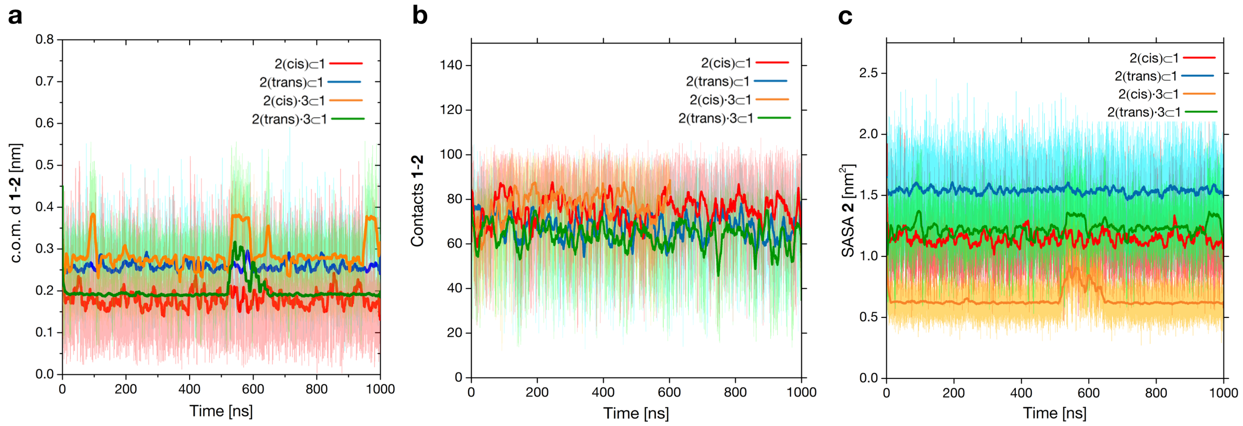


Figure S3: **Time evolution of the different observables analysed in Figure 2 in the main paper.** (**a**) Distance between the geometric center of cage **1** and the center of **2**. (**b**) Contacts between **1** and **2**. (**c**) SASA of **2** in the different complexation conditions. All data reach convergence and equilibration during 1 us of MD simulation.

## Preface on reconstructing dynamics from metadynamics

In these atomistic models, transitions such as, *e.g.*, the isomerization of the amide, as well as amide encapsulation/expulsion in/out the cage are typically rare events, which cannot be effectively sampled *via* classical MD simulation. Recently, it has been demonstrated that the kinetics of rare events can be efficiently reconstructed by means of infrequent WT-MetaD simulations.[28–30] Provided that the the CV and setup of the WT-MetaD simulations are opportunely chosen, this approach relies on the fact that the real unbiased timescale of an event can be retrieved from the statistics obtained for the biased event, by calculating the transition time distributions.

We exploited this approach to calculate the characteristic timescales and the related kinetics of all the key processes studied herein – *i.e.*, the amide hydrolysis rate limiting step, the *cis*-*trans* isomerization of the amide, and its encapsulation/expulsion in/out the cage (details on the WT-MetaD simulations setup for each of these studies are provided in the dedicated sub-sections below).

In particular, the unbiased transition time ($t$) can be calculated from the biased transition time ($t_{WT}$) extracted from each individual infrequent WT-MetaD run (in which the transition is biased/activated) as:

$$t = t_{WT} \langle e^{\beta V(s(\mathbf{R},t))} \rangle_{WT} \tag{S2}$$

where $V(s(\mathbf{R}, t))$ is the time dependent bias used during the simulation, and the exponential is averaged over the WT-MetaD run. The main idea behind this approach is to infrequently deposit the bias onto the free energy landscape to speed-up the transition and to effectively observe it at atomistic resolution during the run. At the same time, the $V(s(\mathbf{R}, t))$ has to be deposited infrequently during the WT-MetaD, in such a way to prevent/minimize the deposition of bias on the transition barrier. The transition times ($t$) estimated from multiple infrequent WT-MetaD runs allow building a transition probability distribution, $P_{n\geq 1}$, defining the probability to effectively observe at least one transition by time $t$:

$$P_{n\geq 1} = 1 - e^{\frac{t}{\tau}} \tag{S3}$$

where $\tau$ is the characteristic timescale expected for unbiased transition. For a rare event, the statistics of transition times fit well with a Poisson distribution.[29,31] The kinetic rate constant ($k$) associated to such rare events can be then estimated from the characteristic transition timescales ($\tau$) as:

$$k = \frac{1}{\tau} \tag{S4}$$

This allows to estimate the unbiased kinetics of a biased transition. The associated transition barrier ($\Delta G^{\ddagger}$), can be then estimated using the Eyring equation:

$$k = \frac{\kappa k_{\mathrm{B}} T}{h} e^{-\frac{\Delta G^{\ddagger}}{RT}} \tag{S5}$$

where $\kappa$ is the transmission coefficient (set to 1 in all cases studied herein, based on the fundamental no-recrossing assumption of transition state theory), $k_B$ is Boltzmann's constant, and $h$ is Planck's constant.

## *Ab initio* metadynamics simulations

*Ab initio* metadynamics simulations have been performed using the CP2K package.[6,32] We employed the semi-empirical density-functional tight-binding (DF-TB) method,[33,34] in its self-consistent charge corrected variant SCC-DFTB.[35] Since our reactions include mostly organic molecules in water, we employed the well validated parametrization set *miomod:nh*.[36] Convergence of the SCF was set at $1.0^{-6}$ Ha. All simulated model systems used for the study of the hydrolysis reaction consist of a cubic box of side length 17.0 Å, containing amide **2** and 160 explicit water molecules, for a total of 510 atoms per simulation cell (Figure S4). Initial minimization and equilibration of this simulation box were performed for the neutral system. Subsequently, the initial configuration for the hydroxide ion was obtained by deleting one

proton from the water molecule closest to the amide bond. The Coordination Number (CN) of the oxygen of the $OH^-$ with respect to the hydrogens in the system was then constrained to be 1, in order to preserve the geometry of an hydroxide ion, following Crespo *et al.*[37] The CN was defined using a Fermi function of the following form: $(1-[r/r_0]^{NN})/(1-[r/r_0]^{ND})$, where $r_0$ = 1.32 Å, $NN$ = 16, and $ND$ = 56 with respect to all of the hydrogens in the system. This CN ensures that the geometry of this species is constrained to be that of the hydroxide ion, which prevents complications arising from the identity of the hydroxide changing due to the Grotthuss mechanism, while still allowing the ion to react with amide.

*Ab initio* metadynamics simulations of the hydroxide ion attack of the amide have been conducted on different amide conformers, having different amide $\omega$ dihedral values which have been kept as contrained with an harmonic potential during the simulations. In particular, having initially set the constraints, every system (containing different $\omega$ amides) has been further thermalized for 3-4 ps. All simulations were run at 333 K in the NVT ensemble using the Canonical Sampling through Velocity Rescaling (CSVR) thermostat.[23]

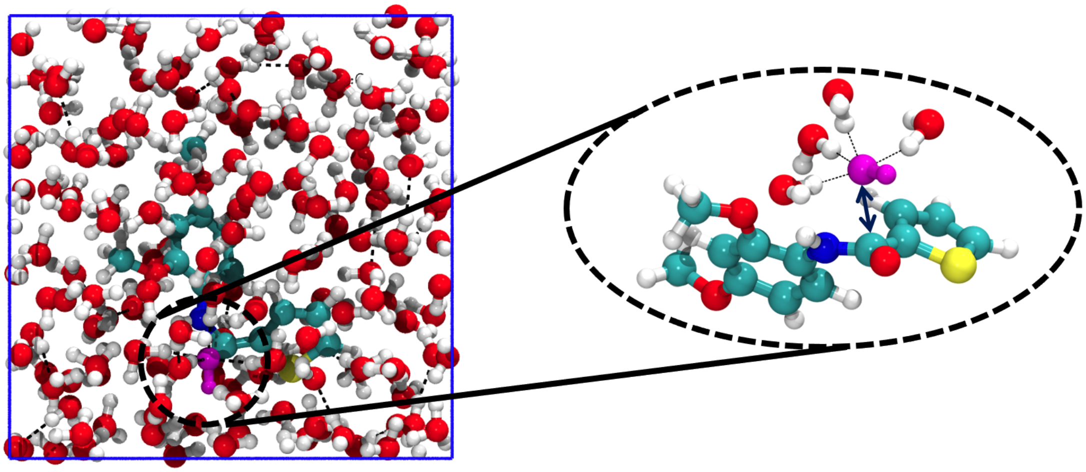

Figure S4: **Ball and stick representation of the equilibrated structure of the amide in a water box with magnification showing the amide, the hydroxyl ion (in magenta) and its solvation shell**.

To estimate the characteristic rates of the $OH^-$ attack/detachment from the carboxyl group of amide **2**, we ran 30 replica *ab initio* infrequent WT-MetaD simulations[38] biasing

respectively the binding/unbinding of the $OH^-$ ion as a function of the $\omega$ dihedral of **2**. In particular, we compared four cases, where the $\omega$ dihedral of amide **2** was constrained to 0, $\pi$, $\pi/4$ and $\pi/2$. From the binding/unbinding transition timescales extracted from the 30 infrequent MetaD runs, we could reconstruct the characteristic unbiased kinetics for the events and estimate the characteristic unbinding/binding timescales, $\tau_{off}$ and $\tau_{on}$ respectively. For all modeled cases, the kinetic constants for hydroxyde ion release from the amide has been calculated as $k_{off} = 1/\tau_{off}$. The kinetic constant for hydroxyde ion attack ($k_{on}$) can be obtained the same way from $\tau_{on}$ – the obtained values have been then corrected considering that our simulation setup is consistent with a hypothetical $OH^-$ concentration equal to the one of pure water (55.6 M), as the $OH^-$ ion starts already (and remains) in close proximity of the amide. We could then estimate the equilibrium constant for the amide hydrolysis ($K$ – see Table S2) as:

$$K = \frac{k_{on}}{k_{off}} \tag{S6}$$

In particular, the case with $\omega$=$\pi/2$ demonstrated to be highly reactive and unstable, proving that this conformer of amide **2** is statistically irrelevant in this case.

In these *ab initio* infrequent WT-MetaD simulations we used the distance between $O_{OH}$ and the C=O carbon as the CV. We deposited hills (initial height = 2.0E-3 Ha) every 100 steps, using a bias-factor of 50. A potential wall was imposed to limit CV values larger than 3 Å. The CV was monitored to determine the transition events, with a cutoff of $<$ 1.4 Å for $\tau_{on}$ and of $>$ 2.8 Å for $\tau_{off}$. Table S2 reports the obtained data for the three dihedral angles and Figure S5 reports the corresponding probability distributions and Poisson fits.

## Conformational metadynamic simulations

In order to characterize the ΔG associated to the *trans*-to-*cis* transition of **2**, we employed WT-MetaD simulations, biasing the 3 dihedral angles – $\omega$, $\psi$ and $\phi$, as described in the

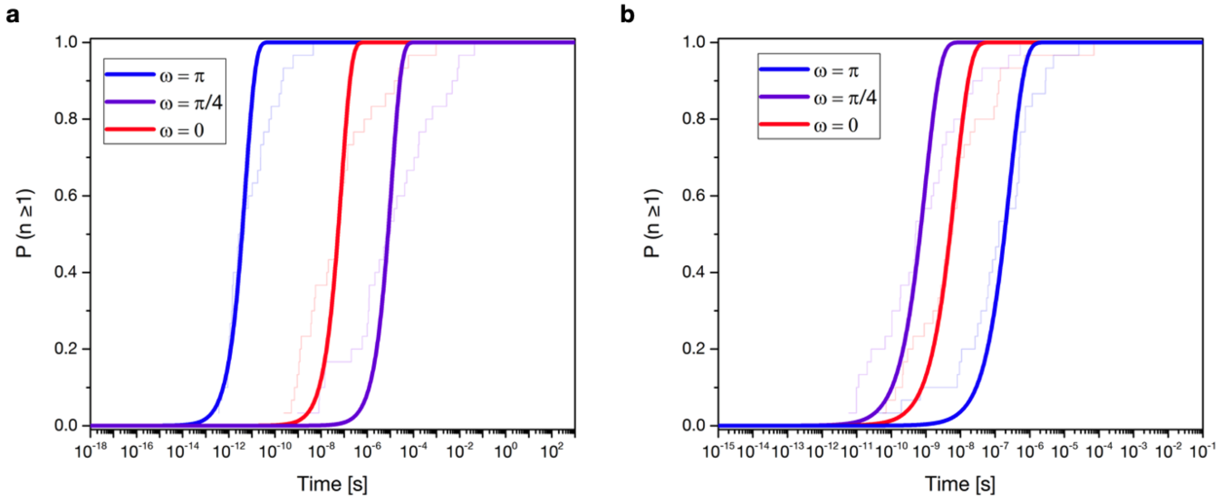


Figure S5: **Estimation of reaction times**. (**a**) Cumulative probability distributions obtained by fitting the hydroxide release times from the TI calculated from the infrequent WT-MetaD simulations for the three values of the $\omega$ dihedral angles $\pi$ (*i.e. trans* conformation), $\pi/4$, 0 (*i.e cis* conformation). From these distributions it is possible to calculate the characteristic times $\tau_{off}$. The $R^2$ for the fittings are, in order, 0.80, 0.69, 0.76. (**b**) Cumulative probability distributions obtained by fitting the hydroxide attach times to generate the TI calculated from the infrequent WT-MetaD simulations for the three values of the $\omega$ dihedral angles $\pi$ (*i.e. trans* conformation), $\pi/4$, 0 (*i.e cis* conformation). From these distributions it is possible to calculate the characteristic times $\tau_{on}$. The $R^2$ for the fittings are, in order, 0.82, 0.76, 0.90.

Table S2: **Reaction equilibrium and kinetic constants**. To each system here studied we report the measured residence times ($\tau_{off}$ and $\tau_{on}$) with the associated asymptotic standard error (%)

, kinetic constants ($k_{off}$ and $k_{on}$), reaction constant ($K_{reac}$) and reactivity score ($\chi_\omega$).

| $\omega$ | $\tau_{off}[s]$ | $\tau_{on}[s]$ | $k_{off}[s^{-1}]$ | $k_{on}[M^{-1}s^{-1}]$ | $K_{reac}[M^{-1}]$ | $\chi_\omega$ |
|---|---|---|---|---|---|---|
| 0 | $7.7 \times 10^{-8}$ (±13.7%) | $7.2 \times 10^{-9}$ (±7.9%) | $1.3 \times 10^{7}$ | $2.5 \times 10^{6}$ | $1.9 \times 10^{-1}$ | $9.0 \times 10^{-4}$ |
| $\pi/4$ | $1.1 \times 10^{-5}$ (±22.5%) | $9.8 \times 10^{-10}$ (±15.4%) | $8.7 \times 10^{4}$ | $1.8 \times 10^{7}$ | $2.1 \times 10^{2}$ | $1.0 \times 10^{0}$ |
| $\pi$ | $5.4 \times 10^{-12}$ (±13.3%) | $2.7 \times 10^{-7}$ (±11.8%) | $1.9 \times 10^{11}$ | $6.7 \times 10^{4}$ | $3.6 \times 10^{-7}$ | $1.7 \times 10^{-9}$ |

main paper – for the four systems, **2** in solution, **2** ⊂ **1**, $\mathbf{2}_2$ ⊂ **1** and **2** · **3** ⊂ **1**. The starting model conformations used in the WT-MetaD simulations of these complexes have been obtained after 1 $\mu$s of preliminary NPT MD equilibration. During this WT-MetaD run, in all simulated systems amide **2** was seen to undergo transition *trans*-to-*cis*, recrossing between the isomers multiple times. For all simulated complexes, we ran at least 1 $\mu$s of WT-MetaD simulation to reach convergence in the calculated *trans*-to-*cis* $\Delta$G. In these WT-MetaD simulations the bias was deposited every 1000 steps (2 ps of simulation time) using Gaussians of initial height of 1.2 kcal/mol, $\sigma$ of 0.33 rad, with bias factor of 25-35 depending on the system.

For the characterization of the barriers and kinetics of the *trans*-to-*cis* isomerization, we turned to infrequent WT-MetaD simulations. We ran 51 infrequent WT-MetaD simulations have been ran for each system where the isomerization of the amide has been activated. In particular, we focused on the isomerization along $\omega$ and $\psi$ dihedral angles for each of our host-guest systems. This allowed us to compare how the encapsulation, and then the molecular crowding inside the cage cavity, affect the dynamics of isomerization of **2**. In these infrequent WT-MetaD runs, the bias was deposited every 5000 steps (10 ps of simulation time) using Gaussians of initial height of 1.2 kcal/mol, $\sigma$ of 0.23 rad, with bias factors between 6-16 depending on the system.

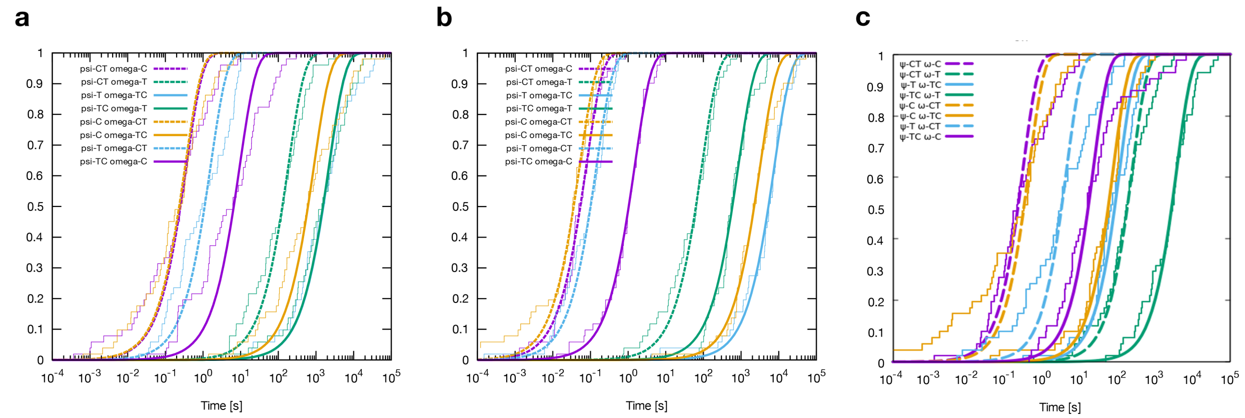


Figure S6: **Estimation of isomerization times**. Cumulative probability distributions obtained by fitting the isomerization times calculated from the infrequent WT-MetaD simulations for the three cases (**a**) amide **2** in solution, (**b**) amide **2** in the cage (**2** ⊂ **1**), (**c**) amide **2** in the cage with co-guest **3** (**2**·**3** ⊂ **1**). We focused on the isomerization along $\omega$ and $\psi$ dihedral angles for each of our host-guest systems: C stands for *cis*, T stands for *trans*, CT means that the *cis*-to-*trans* transition for that angle has been biased in the infrequent WT-MetaD simulation. From these distributions it is possible to calculate the characteristic isomerization times $\tau_{off}$. The average $R^2$ for the fittings was 0.90 and the average asymptotic standard error in the computed isomerization times was 8%.

## Encapsulation/expulsion metadynamics simulations

The isomers of amide **2** show different encapsulation/expulsion kinetics in/out the cage cavity, with different affinities (ΔG), retention times ($\tau$) and characteristic transition rates ($k$). As expected, these quantities are influenced also by the presence of a co-guest (**3**, or another **2**) inside the cavity of **1**. For evaluation of the kinetics constant of the guests encapsulation/expulsion in/out the cage cavity, we conducted multiple infrequent WT-MetaD simulations in which the encapsulations/expulsion of the amide have been activated. In these WT-MetaD simulations, we used as the CVs the contacts between heavy atoms of guest and host-cage (CV1), the host-to-guest center-to-center distance (CV2), and the standard deviation of the contacts between heavy atoms of the guest and of the host-cage (CV3).

We conducted a first explorative WT-MetaD simulation, where the amide was biased to exchange in/out the cage cavity. Qualitatively, similar to what seen also in other host-guest[39] and dynamic supramolecular systems,[40] this simulation showed that the exchange of a guest from the cage cavity into the solvent is most likely a 2-steps process, where (i) the

encapsulated guest is first expelled out of the cage cavity and it remains absorbed onto the cage surface (Figure S7a,b: in-to-out cavity transition), and then (ii), from such absorbed state, it jumps in solution. The same holds for the back (encapsulation) process, but in opposite direction. As explained in the main text, for the purposes of this specific work, the exchange step (i) is the key one. In fact, this transition relates the state where **2** is encapsulated within the cage cavity *vs.* the closest state in free-energy where **2** is out of the cavity of **1** – *i.e.*, the most likely transition (accompanied by the lowest exchange barrier) having consequences in terms of stabilization of the reactive $\mathbf{2}_{cis}$ conformers seen in Figure 3d of the main paper). Indeed, as the crowding has a direct effect on the guest favored conformers and on their related reactivity, being out of the cage and adsorbed on the surface or in solution has negligible effects in terms of crowding for amide.

Reaching a robust convergence in a single WT-MetaD simulation for such a complex multi-step exchange process is prohibitive, and the FES shown in Figure S7a,b has thus just a qualitative/explorative value. As recently done also for other similar systems,[4,41] to characterize the key step (i), we thus opted to reconstruct the guest encapsulation/expulsion exchange profile, the associated ΔG, transition barriers, and characteristic kinetics from multiple infrequent WT-MetaD simulations (see Figure S7c, and Figure 4 in the main paper). In these infrequent WT-MetaD simulations, the bias was deposited every 5000 steps (10 ps of simulation time) using Gaussians of initial height of 0.12 kcal/mol, with bias factor ranging between 10 and 14 (depending on the system) for the expulsion out of the cage cavity, while we used 5 as the bias factor for the WT-MetaD simulations activating the guest encapsulation event. The deposed Gaussians had $\sigma$ equal to 4.0, 0.04 nm, 2.0 along CV1, CV2 and CV3 respectively. For each system, the estimation of the kinetic constants ($k$) for the amide encapsulation/expulsion transitions in/out the cage cavity have been estimated from 50 WT-MetaD simulations. From these, we then could reconstruct all the associated thermodynamic quantities as described in the previous section above (see Equation S2-S5). As demonstrated by the data obtained for all cases, it is clear that in such host-guest

systems the amide guest can be in general considered to be always inside the cage cavity (see Figure S7c, Figure 4 and Table 2 in the main paper) - *i.e.*, in Equation 5 in the main paper $P^{in} \sim 1$ in all simulated cases. Figure S8 reports, for all investigated systems, the probability distributions and Poisson fits for the in-out of cavity exchange process, while Table S3 reports the obtained kinetic quantities, with the associated standard error.

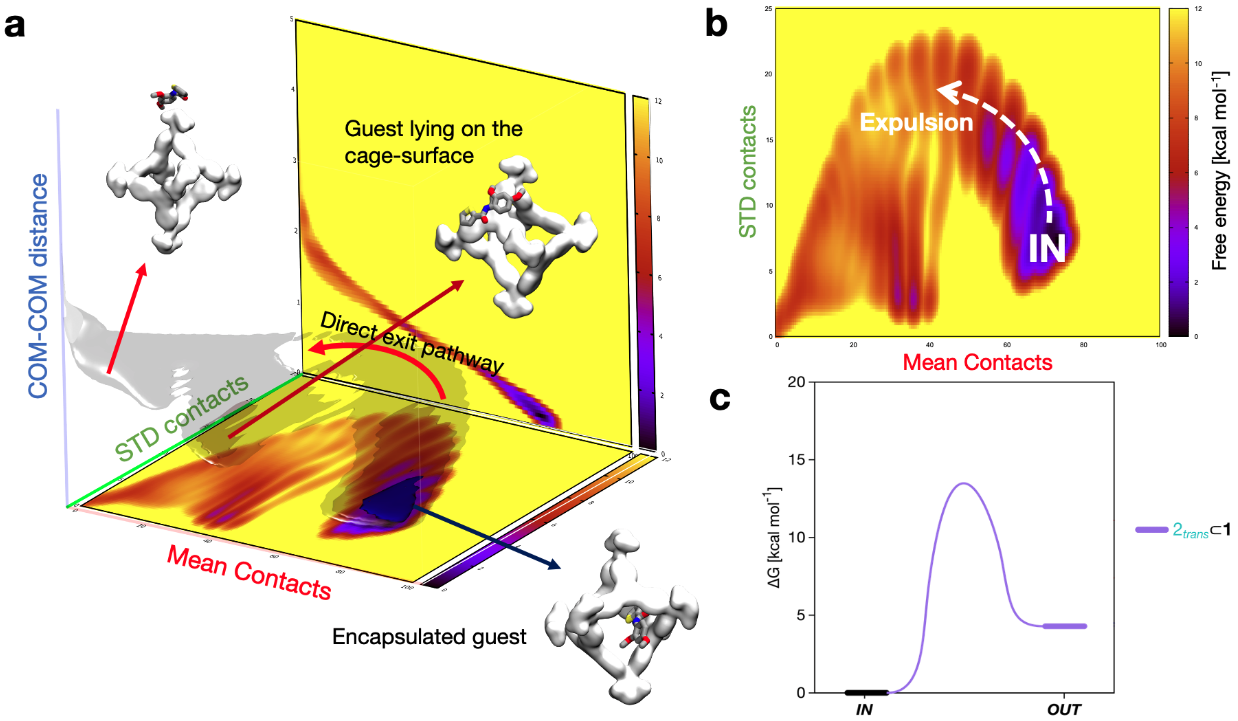


Figure S7: **Explorative metadynamics simulation of $\mathbf{2}_{trans}$ encapsulation/expulsion in/out the cage cavity**. (**a**) Qualitative 3D FES of the guest exchange process (transparent), with 2D projections colored according to the relative free energy in kcal mol$^{-1}$. Selected configuration are reported. (**b**) 2D free energy surface of the in-out of cavity exchange process, projected along 2 of the CVs. (**c**) Guest encapsulation/expulsion exchange free energy profile, including the associated ΔG and transition barrier as obtained from multiple infrequent WT-MetaD simulations, reported for the $\mathbf{2}_{trans}$ case. The other investigated cases are reported in Figure **??** in the main paper.

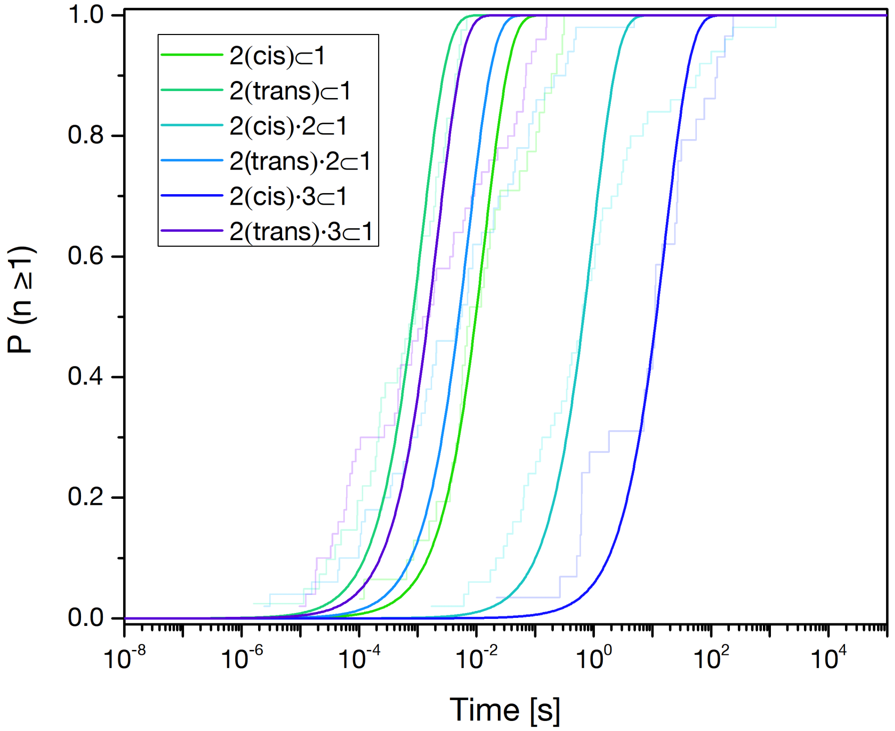


Figure S8: **Estimation of in-out of cavity exchange times**. Cumulative probability distributions obtained by fitting the expulsion times from inside the cage cavity calculated from the infrequent WT-MetaD simulations for the six different encapsulated systems. From these distributions it is possible to calculate the characteristic expulsion times $\tau_{off}$. The $R^2$ for the fittings are, following the order in the legend, 0.90, 0.89, 0.71, 0.85, 0.68, 0.89.

Table S3: **Kinetics of the amide encapsulation/expulsion in/out cavity**. For each simulated host-guest complex, characteristic in-cavity residence times ($t_{off}$) with the associated asymptotic standard error (%), and the associated transition rates ($k_{off}$ and $k_{on}$) estimated from the WT-MetaD simulations are reported.

| Process | $t_{off}$ [s] | k$_{off}$ [s$^{-1}$] | k$_{on}$ [s$^{-1}$] |
|---|---|---|---|
| $\mathbf{2}_{trans} + \mathbf{1} \rightleftarrows \mathbf{2}_{trans} \subset \mathbf{1}$ | $1.2 \times 10^{-3}$ (±6.8%) | $8.3 \times 10^{2}$ | $1.3 \times 10^{6}$ |
| $\mathbf{2}_{cis} + \mathbf{1} \rightleftarrows \mathbf{2}_{cis} \subset \mathbf{1}$ | $1.4 \times 10^{-2}$ (±8.0%) | $7.1 \times 10^{1}$ | $1.8 \times 10^{7}$ |
| $\mathbf{2}_{trans} + \mathbf{2}_{trans} \subset \mathbf{1} \rightleftarrows \mathbf{2}_{trans} \cdot \mathbf{2}_{trans} \subset \mathbf{1}$ | $7.3 \times 10^{-3}$ (±13.8%) | $1.4 \times 10^{2}$ | $3.7 \times 10^{5}$ |
| $\mathbf{2}_{cis} + \mathbf{2}_{trans} \subset \mathbf{1} \rightleftarrows \mathbf{2}_{trans} \cdot \mathbf{2}_{cis} \subset \mathbf{1}$ | $9.6 \times 10^{-1}$ (±8.1%) | $1.0 \times 10^{0}$ | $7.1 \times 10^{6}$ |
| $\mathbf{2}_{trans} + \mathbf{3} \subset \mathbf{1} \rightleftarrows \mathbf{2}_{trans} \cdot \mathbf{3} \subset \mathbf{1}$ | $2.2 \times 10^{-3}$ (±14.4%) | $4.5 \times 10^{2}$ | $1.0 \times 10^{6}$ |
| $\mathbf{2}_{cis} + \mathbf{3} \subset \mathbf{1} \rightleftarrows \mathbf{2}_{cis} \cdot \mathbf{3} \subset \mathbf{1}$ | $1.7 \times 10^{1}$ (±8.4%) | $5.9 \times 10^{-2}$ | $7.7 \times 10^{6}$ |

# References


(1) Wang, J.; Wolf, R. M.; Caldwell, J. W.; Kollman, P. A.; Case, D. A. Development and testing of a general amber force field. *J. Comput. Chem.* **2004**, *25*, 1157–1174.

(2) Seminario, J. M. Calculation of intramolecular force fields from second-derivative tensors. *Int. J Quantum Chem.* **1996**, *60*, 1271–1277.

(3) Li, P.; Merz, K. M. *Structural Genomics*; Springer, 2021; pp 257–275.

(4) Pesce, L.; Perego, C.; Grommet, A. B.; Klajn, R.; Pavan, G. M. Molecular Factors Controlling the Isomerization of Azobenzenes in the Cavity of a Flexible Coordination Cage. *J. Am. Chem. Soc.* **2020**, *142*, 9792–9802.

(5) Bayly, C. I.; Cieplak, P.; Cornell, W.; Kollman, P. A. A well-behaved electrostatic potential based method using charge restraints for deriving atomic charges: the RESP model. *J. Phys. Chem.* **1993**, *97*, 10269–10280.

(6) Hutter, J.; Iannuzzi, M.; Schiffmann, F.; Vandevondele, J. Cp2k: Atomistic simulations of condensed matter systems. *Wiley Interdiscip. Rev. Comput. Mol. Sci.* **2014**, *4*, 15–25.

(7) Becke, A. D. Density-functional thermochemistry. IV. A new dynamical correlation functional and implications for exact-exchange mixing. *J. Chem. Phys.* **1996**, *104*, 1040–1046.

(8) Grimme, S. Semiempirical GGA-type density functional constructed with a long-range dispersion correction. *J. Comp. Chem.* **2006**, *27*, 1787–1799.

(9) Goedecker, S.; Teter, M.; Hutter, J. Separable dual-space Gaussian pseudopotentials. *Phys. Rev. B* **1996**, *54*, 1703.

(10) VandeVondele, J.; Hutter, J. Gaussian basis sets for accurate calculations on molecular systems in gas and condensed phases. *J. Chem. Phys.* **2007**, *127*, 114105.

(11) Wang, J.; Wang, W.; Kollman, P. A.; Case, D. A. Antechamber: an accessory software package for molecular mechanical calculations. *J. Am. Chem. Soc* **2001**, *222*, U403.

(12) Hess, B.; Kutzner, C.; Van Der Spoel, D.; Lindahl, E. GROMACS 4: algorithms for highly efficient, load-balanced, and scalable molecular simulation. *J. Chem. Theory Comput.* **2008**, *4*, 435–447.

(13) Tribello, G. A.; Bonomi, M.; Branduardi, D.; Camilloni, C.; Bussi, G. PLUMED 2: New feathers for an old bird. *Comput. Phys. Commun.* **2014**, *185*, 604 – 613.

(14) Jorgensen, W. L.; Chandrasekhar, J.; Madura, J. D.; Impey, R. W.; Klein, M. L. Comparison of simple potential functions for simulating liquid water. *J. Chem. Phys.* **1983**, *79*, 926–935.

(15) Takezawa, H.; Shitozawa, K.; Fujita, M. Enhanced reactivity of twisted amides inside a molecular cage. *Nat. Chem.* **2020**, *12*, 574–578.

(16) Ramachandran, G.; Mitra, A. K. An explanation for the rare occurrence of cis peptide units in proteins and polypeptides. *J. Mol. Biol.* **1976**, *107*, 85–92.

(17) Craveur, P.; Joseph, A. P.; Poulain, P.; Brevern, A. G. d.; Rebehmed, J. Cis–trans isomerization of omega dihedrals in proteins. *Amino Acids* **2013**, *45*, 279–289.

(18) Drakenberg, T.; Forsén, S. The barrier to internal rotation in monosubstituted amides. *J. Chem. Soc. D* **1971**, 1404–1405.

(19) Weiner, S. J.; Kollman, P. A.; Case, D. A.; Singh, U. C.; Ghio, C.; Alagona, G.; Profeta, S.; Weiner, P. A new force field for molecular mechanical simulation of nucleic acids and proteins. *J. Am. Chem. Soc.* **1984**, *106*, 765–784.

(20) Weiner, S. J.; Kollman, P. A.; Nguyen, D. T.; Case, D. A. An all atom force field for simulations of proteins and nucleic acids. *J. Comput. Chem.* **1986**, *7*, 230–252.

(21) Pitzer, K. S. Potential energies for rotation about single bonds. *Discuss. Faraday Soc.* **1951**, *10*, 66–73.

(22) Doshi, U.; Hamelberg, D. Reoptimization of the AMBER Force Field Parameters for Peptide Bond (Omega) Torsions Using Accelerated Molecular Dynamics. *J. Phys. Chem. B* **2009**, *113*, 16590–16595.

(23) Bussi, G.; Donadio, D.; Parrinello, M. Canonical sampling through velocity rescaling. *J. Chem. Phys.* **2007**, *126*, 014101.

(24) Berendsen, H. J.; Postma, J. v.; van Gunsteren, W. F.; DiNola, A.; Haak, J. R. Molecular dynamics with coupling to an external bath. *J. Chem. Phys.* **1984**, *81*, 3684–3690.

(25) Essmann, U.; Perera, L.; Berkowitz, M. L.; Darden, T.; Lee, H.; Pedersen, L. G. A smooth particle mesh Ewald method. *J. Chem. Phys.* **1995**, *103*, 8577–8593.

(26) Hess, B.; Bekker, H.; Berendsen, H. J. C.; Fraaije, J. G. E. M. LINCS: A linear constraint solver for molecular simulations. *J. Comp. Chem.* **1997**, *18*, 1463–1472.

(27) Samanta, D.; Mukherjee, S.; Patil, Y. P.; Mukherjee, P. S. Self-Assembled Pd6 Open Cage with Triimidazole Walls and the Use of Its Confined Nanospace for Catalytic Knoevenagel- and Diels–Alder Reactions in Aqueous Medium. *Chem. Eur. J.* **2012**, *18*, 12322–12329.

(28) Tiwary, P.; Parrinello, M. From Metadynamics to Dynamics. *Phys. Rev. Lett.* **2013**, *111*, 230602.

(29) Salvalaglio, M.; Tiwary, P.; Parrinello, M. Assessing the reliability of the dynamics reconstructed from metadynamics. *J. Chem. Theory Comput.* **2014**, *10*, 1420–1425.

(30) Bochicchio, D.; Salvalaglio, M.; Pavan, G. M. Into the dynamics of a supramolecular polymer at submolecular resolution. *Nat. Commun.* **2017**, *8*, 1–11.

(31) Resnick, S. I. *Adventures in stochastic processes*; Springer Science & Business Media, 1992.

(32) Kühne, T. D. et al. CP2K: An electronic structure and molecular dynamics software package - Quickstep: Efficient and accurate electronic structure calculations. *J. Chem. Phys.* **2020**, *152*, 194103.

(33) Porezag, D.; Frauenheim, T.; Köhler, T.; Seifert, G.; Kaschner, R. Construction of tight-binding-like potentials on the basis of density-functional theory: Application to carbon. *Phys. Rev. B* **1995**, *51*, 12947–12957.

(34) Seifert, G.; Porezag, D.; Frauenheim, T. Calculations of molecules, clusters, and solids with a simplified LCAO-DFT-LDA scheme. *Int. J. Quantum Chem.* **1996**, *58*, 185–192.

(35) Wu, Y.; Ilie, A.; Crampin, S. Self-consistent charge and dipole density functional tight binding method and application to carbon-based systems. *Comput. Mater. Sci.* **2017**, *134*, 206–213.

(36) Yang,; Yu, H.; York, D.; Cui, Q.; Elstner, M. Extension of the Self-Consistent-Charge Density-Functional Tight-Binding Method: Third-Order Expansion of the Density Functional Theory Total Energy and Introduction of a Modified Effective Coulomb Interaction. *J. Phys. Chem. A* **2007**, *111*, 10861–10873.

(37) Crespo, Y.; Hassanali, A. Characterizing the local solvation environment of OH− in water clusters with AIMD. *J. Chem. Phys.* **2016**, *144*, 074304.

(38) Fleming, K. L.; Tiwary, P.; Pfaendtner, J. New Approach for Investigating Reaction Dynamics and Rates with Ab Initio Calculations. *J. Phys. Chem. A* **2016**, *120*, 299–305.

(39) Norjmaa, G.; Vidossich, P.; Maréchal, J.-D.; Ujaque, G. Modeling Kinetics and Thermo-

dynamics of Guest Encapsulation into the [M4L6]12– Supramolecular Organometallic Cage. *J. Chem. Inf. Model.* **2021**, *61*, 4370–4381.

(40) Bochicchio, D.; Salvalaglio, M.; Pavan, G. M. Into the dynamics of a supramolecular polymer at submolecular resolution. *Nat. Commun.* **2017**, *8*, 147.

(41) Bochicchio, D.; Kwangmettatam, S.; Kudernac, T.; Pavan, G. M. How Defects Control the Out-of-Equilibrium Dissipative Evolution of a Supramolecular Tubule. *ACS Nano* **2019**, *13*, 4322–4334.
